# *Arsenophonus* and *Sodalis* replacements shape evolution of symbiosis in louse flies

**DOI:** 10.7717/peerj.4099

**Published:** 2017-12-11

**Authors:** Eva Šochová, Filip Husník, Eva Nováková, Ali Halajian, Václav Hypša

**Affiliations:** 1 Department of Parasitology, University of South Bohemia, České Budějovice, Czech Republic; 2 Department of Molecular Biology, University of South Bohemia, České Budějovice, Czech Republic; 3 Institute of Parasitology, Biology Centre, Czech Academy of Sciences, České Budějovice, Czech Republic; 4 Department of Biodiversity, University of Limpopo, Sovenga, South Africa

**Keywords:** *Arsenophonus*, *Sodalis*, *Wolbachia*, Louse flies, Replacements, Phylogeny

## Abstract

Symbiotic interactions between insects and bacteria are ubiquitous and form a continuum from loose facultative symbiosis to greatly intimate and stable obligate symbiosis. In blood-sucking insects living exclusively on vertebrate blood, obligate endosymbionts are essential for hosts and hypothesized to supplement B-vitamins and cofactors missing from their blood diet. The role and distribution of facultative endosymbionts and their evolutionary significance as seeds of obligate symbioses are much less understood. Here, using phylogenetic approaches, we focus on the Hippoboscidae phylogeny as well as the stability and dynamics of obligate symbioses within this bloodsucking group. In particular, we demonstrate a new potentially obligate lineage of *Sodalis* co-evolving with the Olfersini subclade of Hippoboscidae. We also show several likely facultative *Sodalis* lineages closely related to *Sodalis praecaptivus* (HS strain) and suggest repeated acquisition of novel symbionts from the environment. Similar to *Sodalis*, *Arsenophonus* endosymbionts also form both obligate endosymbiotic lineages co-evolving with their hosts (Ornithomyini and Ornithoica groups) as well as possibly facultative infections incongruent with the Hippoboscidae phylogeny. Finally, we reveal substantial diversity of *Wolbachia* strains detected in Hippoboscidae samples falling into three supergroups: A, B, and the most common F. Altogether, our results prove the associations between Hippoboscoidea and their symbiotic bacteria to undergo surprisingly dynamic, yet selective, evolutionary processes strongly shaped by repeated endosymbiont replacements. Interestingly, obligate symbionts only originate from two endosymbiont genera, *Arsenophonus* and *Sodalis*, suggesting that the host is either highly selective about its future obligate symbionts or that these two lineages are the most competitive when establishing symbioses in louse flies.

## Background

Symbiotic associations are widespread among animals and bacteria and often considered to undergo a common evolution as a holobiont (Zilber-Rosenberg & Rosenberg, 2008). The host and symbiont are either fully dependent on each other for reproduction and survival (obligate symbiosis) or not (facultative symbiosis), but in reality there is a gradient of such interactions (Moran, McCutcheon & Nakabachi, 2008). Any establishment of a symbiotic association brings not only advantages, but also several challenges to both partners. Perhaps the most crucial is that after entering the host, the endosymbiont genome tends to decay due to population genetic processes affecting asexual organisms with small effective population sizes (Moran, 1996) and the host is becoming dependent on such a degenerating symbiont (Koga et al., 2007; Pais et al., 2008). Since symbionts are essential for the host, the host can try to escape from this evolutionary ‘rabbit hole’ by an acquisition of novel symbionts or via endosymbiont replacement and supplementation (Bennett & Moran, 2015). This phenomenon, known in almost all insect symbiotic groups, was especially studied in the sap-feeding group Hemiptera (Sudakaran, Kost & Kaltenpoth, 2017), while only few studies were performed from blood-sucking groups.

Blood-sucking insects, living exclusively on vertebrate blood, such as sucking lice (Allen et al., 2007; Hypša & Křížek, 2007; Fukatsu et al., 2009; Allen et al., 2016), bed bugs (Hypša & Aksoy, 1997; Hosokawa et al., 2010; Nikoh et al., 2014), kissing bugs (Ben-Yakir, 1987; Beard et al., 1992; Hypša & Dale, 1997; Šorfová, Škeříková & Hypša, 2008; Pachebat et al., 2013), tsetse flies (Aksoy, 1995; Dale & Maudlin, 1999), bat flies (Trowbridge, Dittmar & Whiting, 2006; Hosokawa et al., 2012; Wilkinson et al., 2016), and louse flies (Trowbridge, Dittmar & Whiting, 2006; Nováková & Hypša, 2007; Chrudimský et al., 2012) have established symbiotic associations with bacteria from different lineages, mostly α-proteobacteria (Hosokawa et al., 2010) and γ-proteobacteria (Aksoy, 1995; Hypša & Aksoy, 1997; Hypša & Dale, 1997; Dale et al., 2006; Allen et al., 2007; Hypša & Křížek, 2007; Nováková & Hypša, 2007; Chrudimský et al., 2012; Hosokawa et al., 2012; Wilkinson et al., 2016). Obligate symbionts of these blood-sucking hosts are hypothesized to supplement B-vitamins and cofactors missing from their blood diet or present at too low concentration (Akman et al., 2002; Kirkness et al., 2010; Rio et al., 2012; Nikoh et al., 2014; Nováková et al., 2015; Boyd et al., 2016; Boyd et al., 2017), but experimental evidence supporting this hypothesis is scarce (Hosokawa et al., 2010; Nikoh et al., 2014; Michalkova et al., 2014; Snyder & Rio, 2015). The role played by facultative bacteria in blood-sucking hosts is even less understood, with metabolic or protective function as the two main working hypotheses (Geiger et al., 2005; Geiger et al., 2007; Toh et al., 2006; Belda et al., 2010; Snyder et al., 2010; Weiss et al., 2013).

Due to their medical importance, tsetse flies (Diptera, Glossinidae) belong to the most frequently studied models of such symbioses (International Glossina Genome Initiative, 2014). They harbour three different symbiotic bacteria: obligate symbiont *Wigglesworthia glossinidia* which is essential for the host survival (Pais et al., 2008), facultative symbiont *Sodalis glossinidius* which was suggested to cooperate with *Wigglesworthia* on thiamine biosynthesis (Belda et al., 2010), and reproductive manipulator *Wolbachia* (Pais et al., 2011). Considerable amount of information has till now been accumulated on the distribution, genomics and functions of these bacteria (Akman et al., 2002; Toh et al., 2006; Rio et al., 2012; Balmand et al., 2013; Michalkova et al., 2014; Snyder & Rio, 2015). In contrast to our understanding of tsetse fly symbioses, only scarce data are available on the symbioses in its closely related groups. Apart from Glossinidae, the superfamily Hippoboscoidea includes additional three families of obligatory blood-sucking flies, tightly associated with endosymbionts, namely Nycteribiidae, Streblidae, and Hippoboscidae. Monophyly of Hippoboscoidea has been confirmed by numerous studies (Nirmala, Hypša & Žurovec, 2001; Dittmar et al., 2006; Petersen et al., 2007; Kutty et al., 2010), but its inner topology has not been fully resolved. The monophyletic family Glossinidae is considered to be a sister group to the three remaining families together designated as Pupipara (Petersen et al., 2007). The two groups associated with bats probably form one branch, where Nycteribiidae seems to be monophyletic while monophyly of Streblidae was not conclusively confirmed (Dittmar et al., 2006; Petersen et al., 2007; Kutty et al., 2010). According to several studies, Hippoboscidae is regarded to be a monophyletic group with not well-resolved exact position in the tree (Nirmala, Hypša & Žurovec, 2001; Dittmar et al., 2006; Petersen et al., 2007). However, louse flies were also shown to be paraphyletic in respect to bat flies (Dittmar et al., 2006; Kutty et al., 2010).

Nycteribiidae, Streblidae (bat flies), and Hippoboscidae (louse flies) are often associated with *Arsenophonus* bacteria (Trowbridge, Dittmar & Whiting, 2006; Dale et al., 2006; Nováková, Hypša & Moran, 2009; Morse et al., 2013; Duron et al., 2014). In some cases, these symbionts form clades of obligate lineages coevolving with their hosts, but some of *Arsenophonus* lineages are likely representing loosely associated facultative symbionts spread horizontally across the population (Nováková, Hypša & Moran, 2009; Morse et al., 2013; Duron et al., 2014). Bat flies and louse flies are also commonly infected with *Bartonella* spp. (Halos et al., 2004; Morse et al., 2012b). *Wolbachia* infection was found in all Hippoboscoidea groups (Pais et al., 2011; Hosokawa et al., 2012; Morse et al., 2012a; Nováková et al., 2015). Moreover, several Hippoboscidae species were also found to harbour distinct lineages of *Sodalis*-like bacteria (Dale et al., 2006; Nováková & Hypša, 2007; Chrudimský et al., 2012) likely representing similar facultative-obligatory gradient of symbioses as observed for *Arsenophonus*.

Hippoboscoidea thus represent a group of blood-sucking insects with strikingly dynamic symbioses. Obligate symbionts from *Arsenophonus* and *Sodalis* clades tend to come and go, disrupting the almost flawless host-symbiont co-phylogenies often seen in insect-bacteria systems. However, why are the endosymbiont replacements so common and what keeps the symbiont consortia limited to the specific bacterial clades remains unknown. Tsetse flies as medically important vectors of pathogens are undoubtedly the most studied Hippoboscoidea lineage. However, their low species diversity (22 species), sister relationship to all other clades, and host specificity to mammals, do not allow to draw any general conclusions about the evolution of symbiosis in Hippoboscoidea. To fully understand the symbiotic turn-over, more attention needs to be paid to the neglected Nycteriibidae, Streblidae, and Hippoboscidae lineages. Here, using gene sequencing and draft genome data from all involved partners, we present phylogenies of Hippoboscidae and their symbiont lineages and try to untangle their relationship to the host. In particular, we ask if these are obligate co-evolving lineages, facultative infections, or if they likely represent recent symbiont replacements just re-starting the obligate relationship.

## Methods

### Sample collection and DNA isolation

Samples of louse flies were collected in seven countries (South Africa, Papua New Guinea, Ecuador—Galapagos, Vietnam, France, Slovakia, and the Czech Republic; see Table S1 for details), the single sample of bat fly was collected in the Czech Republic. All samples were stored in 96% ethanol at −20 °C. DNA was extracted using the QIAamp DNA Micro Kit (Qiagen, Hilden, Germany) according to the manufacturer’s protocol. DNA quality was verified using the Qubit High Sensitivity Kit (Invitrogen) and 1% agarose gel electrophoresis.

### PCR, cloning, and sequencing

All DNA samples were used for amplification of three host genes (COI, 16S rRNA gene, EF) and symbiont screening with 16S rRNA gene primers (Table S2). Ten *Wolbachia* positive samples were used for MLST typing (*cox*A, *fbp*A, *fts*Z, *gat*B, *hcp*A; see Table S2). PCR reaction was performed under standard conditions using High Fidelity PCR Enzyme Mix (Thermo Scientific, Waltham, MA, USA) and Hot Start Tag DNA Polymerase (Qiagen, Hilden, Germany) according to the manufacturer’s protocol. PCR products were analysed using 1% agarose gel electrophoresis and all symbiont 16S rDNA products were cloned into pGEM^®^–T Easy vector (Promega, Madison, WI, USA) according to the manufacturer’s protocol. Inserts from selected colonies were amplified using T7 and SP6 primers or isolated from plasmids using the Jetquick Plasmid Miniprep Spin Kit (Genomed GmbH, Löhne, Germany). Sanger sequencing was performed by an ABI Automatic Sequencer 3730XL (Macrogen Inc., Geumchun-gu-Seoul, Korea) or ABI Prism 310 Sequencer (SEQme, Dobříš, the Czech Republic).

In addition to sequencing, we also included in our analyses genomic data of *Melophagus ovinus* (Nováková et al., 2015), *Lipoptena cervi* (Nováková et al., 2016), *Ornithomya biloba*, and *Crataerina pallida* (Figshare: https://figshare.com/s/e1488900c5cb62af69ab) as well as their endosymbionts (see Table S1).

Although there is MLST available for *Arsenophonus* bacteria (Duron, Wilkes & Hurst, 2010), we were not successful in amplifying these genes.

### Alignments and phylogenetic analyses

The assemblies of raw sequences were performed in Geneious v8.1.7 (Kearse et al., 2012). Datasets were composed of the assembled sequences, extracted genomic sequences, sequences downloaded from GenBank (see Table S4) or the *Wolbachia* MLST database. The sequences were aligned with Mafft v7.017 (Katoh, 2002; Katoh, Asimenos & Toh, 2009) implemented in Geneious using an E-INS-i algorithm with default parameters. The alignments were not trimmed as trimming resulted in massive loss of informative position. Phylogenetic analyses were carried out using maximum likelihood (ML) in PhyML v3.0 (Guindon & Gascuel, 2003; Guindon et al., 2009) and Bayesian inference (BI) in MrBayes v3.1.2 (Huelsenbeck & Ronquist, 2001). The *GTR* + *I* + Γ evolutionary model was selected in jModelTest (Posada, 2009) according to the Akaike Information Criterion (AIC). The subtree prunning and regrafting (SPR) tree search algorithm and 100 bootstrap pseudoreplicates were used in the ML analyses. BI runs were carried out for 10 million generations with default parameters, and Tracer v1.6 (http://tree.bio.ed.ac.uk/software/tracer/) was used for convergence and burn-in examination. Phylogenetic trees were visualised and rooted in FigTree v1.4.2 (http://tree.bio.ed.ac.uk/software/figtree/) and their final graphical adjustments were performed in Inkscape v0.91 (https://inkscape.org/en/).

Host phylogeny was reconstructed using single-gene analyses and a concatenated matrix of three genes (mitochondrial 16S rRNA, mitochondrial cytochrome oxidase I, and nuclear elongation factor). Concatenation of genes was performed in Phyutility 2.2.6 (Smith & Dunn, 2008). Phylogenetic trees were inferred for all species from the Hippoboscoidea superfamily, as well as for smaller datasets comprising only Hippoboscidae species. This approach was employed to reveal possible artefacts resulting from missing data and poor taxon-sampling (e.g., short, ∼360 bp, sequences of COI available for Streblidae and Nycteribiidae).

### Mitochondrial genomes

Problems with reconstruction of host phylogeny based on mitochondrial genes (16S and COI) lead us to assemble mitochondrial genomes of four main louse fly lineages. Contigs of mitochondrial genomes were identified in genomic data of *M. ovinus*, *L. cervi*, *O. biloba*, and *C. pallida* using BLASTn and tBLASTn searches (Altschul et al., 1990). Open reading frame identification and preliminary annotations were performed using NCBI BlastSearch in Geneious. For identification of Numts, raw sequences were mapped to mitochondrial data using Bowtie v2.2.3 (Langmead & Salzberg, 2012). Web annotation server MITOS (http://mitos.bioinf.uni-leipzig.de/) was used for final annotation of proteins and rRNA/tRNA genes. We selected 15 mitochondrial genes (Table S4) present in all included taxa for phylogenetic inference as described above.

## Results

### Phylogenetic data

We obtained 138 host sequences: 31 sequences of 16S rRNA of 208–567 bp, 48 sequences of EF of 280–890 bp, and 59 sequences of COI of 299–1,491 bp; and 70 symbiont 16S rRNA sequences of 269–1,210 bp. We also assembled and annotated 4 host mitochondrial genomes of 15,975–16,445 bp. For more details see Table S3 . All raw sequences can be found online in Data S1 (their description is included in Table S6).

### Hippoboscidae phylogeny

**Figure 1:**
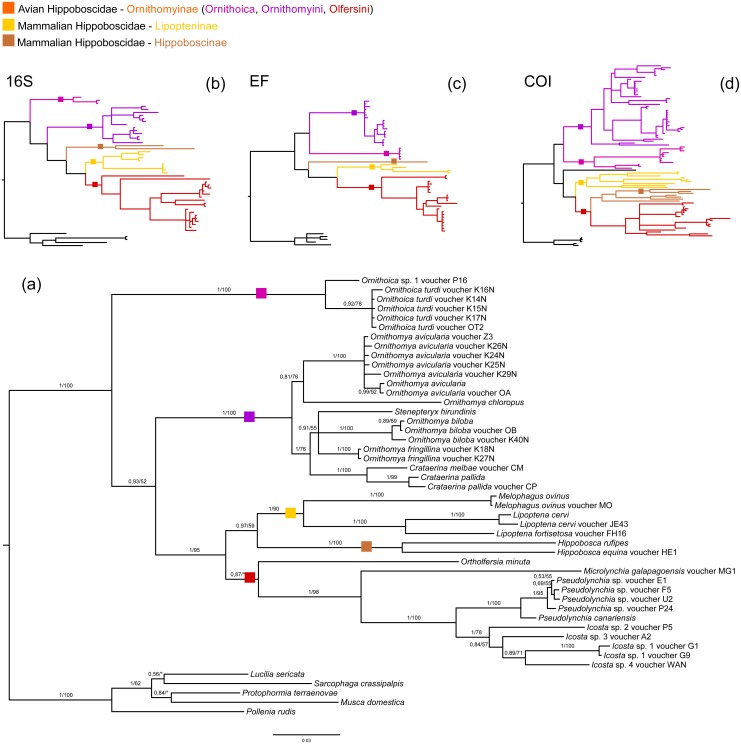
Host phylogeny. (A) Host phylogeny derived from concatenation of three genes: 16S rRNA, EF, and COI. The phylogeny was reconstructed by BI analysis. Posterior probabilities and bootstrap support are printed upon branches, respectively (asterisk was used for very low or missing bootstrap branch support). Taxa labelled with voucher are newly sequenced in this study. Genomic COI sequences are labelled with rRNA. Three smaller trees on the top of the figure represent outlines of three separate phylogenetic trees based on BI analyses of 16S rRNA (B), EF (C), and COI (D) genes. Full versions of these phylogenies are included in Figs. S6–S8. Three main families of Hippoboscidae are colour coded: yellow for Lipopteninae (one group), brown for Hippoboscinae (one group), and orange for Ornithomiinae (three groups). Colour squares label branches where are placed main Hippoboscidae groups. This labelling corresponds with labelling of branches at smaller outlines, which are in addition to this highlighted with the same colour. All host trees are included in Figs. S1–S8.

We reconstructed host phylogeny using three markers: 16S rRNA, EF and COI; as well as mitochondrial genomes. Our analyses of draft genome data revealed that all analysed mitochondrial genomes of louse flies are also present as Numts (nuclear mitochondrial DNA) on the host chromosomes, especially the COI gene often used for phylogenetic analyses. The taxonomically restricted mitochondrial genome matrix verified monophyly of Hippoboscoidea (Fig. S1). Our three-gene dataset yielded only partially resolved and unstable inner Hippoboscoidea phylogeny. Glossinidae and Nycteribiidae formed a well-defined monophyletic groups (only ML analysis of COI did not confirm monophyly of Nycteribiidae and also did not resolve its relationship to Streblidae), but monophyly of Hippoboscidae and Streblidae was not well supported and different genes/analyses frequently inferred contradictory topologies. Within Hippoboscidae, the position of the Hippoboscinae group and the genus *Ornithoica* were the most problematic (Fig. 1, Figs. S2–S8).

### *Arsenophonus* and *Sodalis* phylogenies

In total, 70 endosymbiont 16S rRNA genes were sequenced in this study and six additional sequences of this gene were mined from our draft genomic data: four of *Arsenophonus*, one of *Sodalis*, and one of *Wolbachia*. Twenty-nine symbionts were identified as members of the genus *Arsenophonus*, 13 symbionts were the most similar to *Sodalis*-allied species, and 28 sequences were of *Wolbachia* origin. Despite cloning, we did not obtain any sequences of *Bartonella* reported to occur in some Hippoboscoidea. Moreover, using only phylogenetic approach, we would not be able to decide whether *Bartonella*-Hippoboscidae interaction is mutualistic or pathogenic, therefore *Bartonella* symbiosis is not in the scope of this manuscript. Putative assignment to the obligate or likely facultative symbiont categories was based on GC content of their 16S rRNA gene and genomic data available (Table S3), branch length, and the phylogenetic analyses.

**Figure 2:**
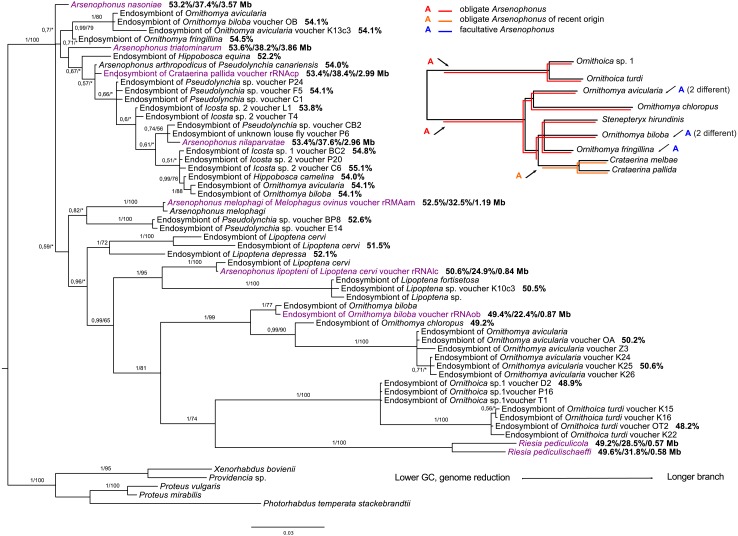
16S rRNA phylogeny of *Arsenophonus* in Hippoboscidae inferred by BI analysis. Posterior probabilities and bootstrap support are printed upon branches, respectively (asterisk was used for very low or missing bootstrap branch support). Taxa labelled with voucher are newly sequenced in this study. Genomic sequences are labelled with rRNA. Taxa in dark purple represent *Arsenophonus* bacteria which genome was sequenced. Numbers behind these taxa correspond to their GC content of 16S rRNA, GC content of genome, and genome size, respectively. Numbers behind other taxa correspond to GC content of their 16S rRNA. The smaller picture on the right side represents host phylogeny to which symbiont phylogeny was compared. Red lineages correspond to obligate symbionts while orange lineage is symbiont of recent origin. The blue A represent likely facultative *Arsenophonus* infection. To achieve this, we also used the information available on *gro*EL gene by Morse et al. (2013) and Duron et al. (2014). Phylogenetic reconstructions of *Arsenophonus* of entire Hippoboscoidea and all *Arsenophonus* bacteria are included in Figs. S9 and S10.

Phylogenetic analyses of the genus *Arsenophonus* based on 16S rDNA sequences revealed several distinct clades of likely obligate *Arsenophonus* species congruent with their host phylogeny, partially within the Nycteribiidae, Streblidae, and several Hippoboscidae lineages (Fig. 2, Figs. S9 and S10). However, it is important to note that these clades do not form a single monophyletic clade of co-diverging symbionts, but rather several separate lineages. Within Hippoboscidae, the *Arsenophonus* sequences from the Ornithomyini group form a monophyletic clade congruent with Ornithomyini phylogeny except *Arsenophonus* symbiont of *Crataerina* spp. which was probably recently replaced by another *Arsenophonus* bacteria. Other obligate *Arsenophonus* lineages were detected in the genera *Lipoptena*, *Melophagus*, and *Ornithoica*. All other *Arsenophonus* sequences from the Hippoboscidae either represent facultative symbionts or putatively obligate symbioses which are impossible to reliably detect by phylogenetic methods (but see the discussion for *Hippobosca* sp.).

Most of the putatively facultative endosymbionts of the Hippoboscidae typically possess short branches and are also related with the previously described species *Arsenophonus arthropodicus* and *Arsenophonus nasoniae*. Interestingly, both obligate and likely facultative lineages were detected from several species, e.g., *Ornithomya biloba*, *Ornithomya avicularia*, and *Ornithomya fringillina* (Fig. 2). Phylogenetic analyses including symbionts from the genera *Nycterophylia* and *Trichobius* did not clearly place them into the *Arsenophonus* genus. Rather, they likely represent closely related lineages to the *Arsenophonus* clade as their position was unstable and changed with different taxon samplings and methods.

**Figure 3:**
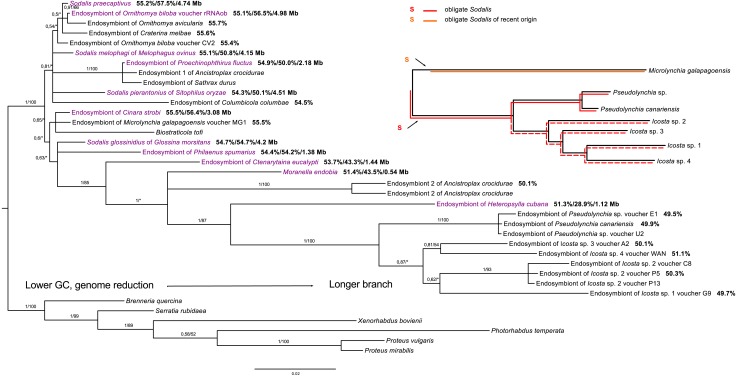
16S rRNA phylogeny of Sodalis in Hippoboscidae inferred by BI analysis. Posterior probabilities and bootstrap support are printed upon branches, respectively (asterisk was used for very low or missing bootstrap branch support). Taxa labelled with voucher are newly sequenced in this study. Taxa in dark purple represent *Sodalis*-like bacteria which genome was sequenced. Numbers behind these taxa correspond to their GC content of 16S rRNA, GC content of genome, and genome size, respectively. Numbers behind other taxa correspond to GC content of their 16S rRNA. Red lineages correspond to obligate symbionts while orange lineage is symbiont of recent origin. The red dashed line shows that co-evolution between *Icosta* spp. and their obligate endosymbiont imperfect.

Within *Sodalis*, the phylogenetic reconstruction revealed a putatively obligate endosymbiont from the tribe Olfersini, including the genera *Pseudolynchia* and *Icosta*, and several facultative lineages. However, co-evolution with *Icosta* sp. seems to be imperfect and does not strictly follow the host phylogeny (Fig. 3).

### *Wolbachia* MLST analysis

**Figure 4:**
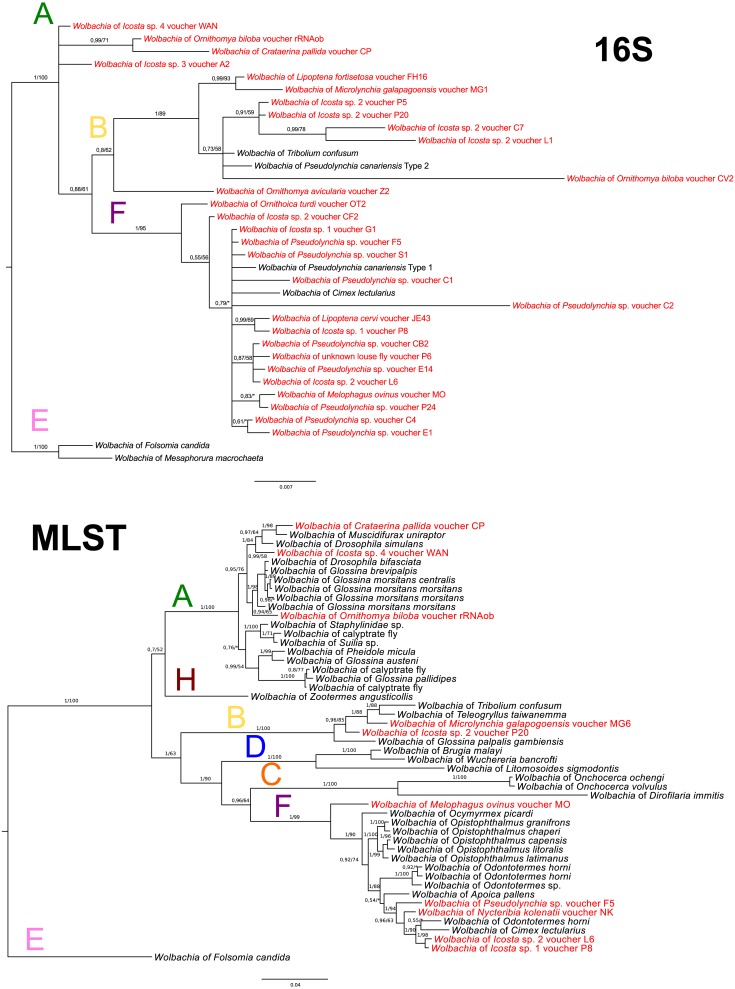
*Wolbachia* phylogeny. (A) *Wolbachia* phylogeny inferred from 16S rRNA by BI analysis. (B) *Wolbachia* phylogeny inferred from MLST genes by BI analysis. Posterior probabilities and bootstrap support are printed upon branches, respectively (asterisk was used for very low or missing bootstrap branch support). Colour letters upon branches correspond to *Wolbachia* supergroups. Taxa in red represent *Wolbachia* bacteria from Hippoboscidae and Nycteribidae which are newly sequenced in this study. Taxa labelled with # in the 16S tree represent taxa which were used for the MLST analysis. *Wolbachia* from *O*. *biloba*, which was obtained from genomic data, is labelled with rRNAob. Supergroup E was used for rooting both trees.

In *Wolbachia*, the 16S rDNA sequences were used only for an approximate supergroup determination (Fig. 4A). The MLST analysis was performed with ten selected species (one of them was obtained from genomic data of *O*. *biloba*; see Table S3). Overall prevalence of *Wolbachia* in louse flies is 54.55%; 30 positive individuals out of 55 diagnosed. The supergroup A was detected from 4 species (4 individuals), the supergroup B from 5 species (9 individuals), and the supergroup F from 7 species (17 individuals) (Figs. 4A–4B). Additionally, *Nycteribia kolenatii* (one individual) was infected with the supergroup F.

## Discussion

### Hippoboscidae phylogeny: an unfinished portrait

Although closely related to the medically important tsetse flies, the other hippoboscoids have only rarely been studied and their phylogeny is still unclear. Based on our concatenated matrix, we obtained the topology which to some extent resembles the one presented Petersen et al. (2007), although with slightly different taxon sampling (Fig. 1; Fig. S2). However, our three single-gene datasets implied only poor phylogenetic signal available carried by the hippoboscoid sequences. Therefore, we took an advantage of the four complete mitochondrial genomes reconstructed in this study to test the reliability of the previous phylogenetic reconstructions. The phylogenetic reconstruction based on the mitochondrial matrix correspond to the three-gene concatenated matrix phylogeny suggesting that mitochondrial genomes would be valuable for further phylogenetic analyses of this group (Fig. 1; Fig. S1). According to our results, Glossinidae, Nycteribiidae and Hippoboscidae were retained as monophyletic groups, but monophyly of Streblidae was not supported using the complete matrix (Fig. S2). Streblidae lineage appears to be paraphyletic with respect to Nycteribiidae and clusters into two groups, the Old World and the New World species, as previously reported (Dittmar et al., 2006; Kutty et al., 2010). Within Hippoboscidae, the groups Lipopteninae, Hippoboscinae, Ornithomyini and Olfersini (nomenclature was adopted from Petersen et al. (2007)) are well-defined and monophyletic, but their exact relationships are still not clear. The most problematic taxa are Hippoboscinae and also the genus *Ornithoica* with their positions depending on the used genes/analyses (Fig. 1; Figs. S2–S8). A possible explanation for these inconsistencies in the topologies can be a hypothetical rapid radiation from the ancestor of Hippoboscoidea group into main subfamilies of Hippoboscidae leaving in the sequences only very weak phylogenetic signal for this period of Hippoboscidae evolution. The most difficulties in reconstructing Hippoboscoidea phylogeny is caused by missing data (only short sequences of COI are available especially for Nycteribiidae and Streblidae in the GenBank; Fig. S3). Moreover, COI phylogenies are known to be affected by numerous pseudogenes called Numts (Black IV & Bernhardt, 2009). The Numts, we found to be common in louse fly genomes, can thus also contribute to the intricacy of presented phylogenies. On the other hand, EF seems to provide plausible phylogenetic information (Fig. S4). The biggest drawback of this marker however lies in the data availability in public databases, restricting an appropriate taxon sampling for the Hippoboscoidea superfamily.

### Hidden endosymbiont diversity within the Hippoboscidae family

Among the three most commonly detected Hippoboscidae endosymbionts, attention has been predominantly paid to *Arsenophonus* as the supposedly most common obligate endosymbiont of this group. Our data show that several different lineages of *Arsenophonus* have established the symbiotic lifestyle within Hippoboscidae (Fig. 2). According to our results supported by genomic data, there are at least four lineages of likely obligate endosymbionts: *Arsenophonus* in Ornithomyini (genomes of *Arsenophonus* from *Ornithomya biloba* and *Crataerina pallida* will be published elsewhere), *Arsenophonus* in *Ornithoica* spp., previously described *Arsenophonus melophagi* (Nováková et al., 2015) and *Arsenophonus lipopteni* (Nováková et al., 2016). All these possess reduced genomes with low GC content as a typical feature of obligate endosymbionts (McCutcheon & Moran, 2012). Interestingly, within Ornithomyini, the original obligate *Arsenophonus* endosymbiont of *Crataerina* spp. was recently replaced by another *Arsenophonus* bacterium with ongoing genome reduction (Figshare: https://figshare.com/s/e1488900c5cb62af69ab). Apart from these potentially obligate lineages, there are other hippoboscid associated *Arsenophonus* bacteria distributed in the phylogenetic tree among *Arsenophonus* endosymbionts with likely facultative or free-living lifestyle (Fig. S10). This pattern suggests *Arsenophonus* is likely being repeatedly acquired from the environment. It has been hypothesized that obligate endosymbionts often evolve from facultative symbionts which are no longer capable of horizontal transmission between the hosts (Moran, McCutcheon & Nakabachi, 2008). Due to their recent change of lifestyle, endosymbionts with an ongoing genome reduction in many ways resemble facultative symbionts, e.g., their positions in phylogenetic trees are not stable and differ with the analysis method and taxon sampling (Fig. 2, Figs. S9 and S10). Such nascent stage of endosymbiosis was indicated for the obligate *Arsenophonus* endosymbiont of *C*. *pallida* (Figshare: https://figshare.com/s/e1488900c5cb62af69ab) and similar results can be expected for *Arsenophonus* endosymbionts of *Hippobosca* species.

Within bat flies, we found obligate *Arsenophonus* lineages in both Nycteribiidae and Streblidae as well as several presumably facultative *Arsenophonus* infections in both groups (Figs. S9 and S10). Similar results were reported in several previous studies (Morse et al., 2013; Duron et al., 2014; Wilkinson et al., 2016). Members of the *Arsenophonus* clade were also reported from Nycterophyliinae and Trichobiinae (Streblidae) (Morse et al., 2012a) and *Cyclopodia dubia* (Nycteribiidae) (Wilkinson et al., 2016). However, our results do not support their placement within the clade, as these sequences were attracted by the long branches in the ML analyses. The endosymbiont of Nycterophyliinae and Trichobiinae probably represents an ancient lineage closely related to *Arsenophonus* clade (Fig. S9) while the endosymbiont of *Cyclopodia dubia* is more likely related with *Pectobacterium* spp.; therefore, we excluded this bacterium from our further analyses. These findings indicate that bat flies established the endosymbiotic lifestyle several times independently with at least three bacterial genera.

In contrast to *Arsenophonus*, only a few studies reported *Sodalis*-like endosymbiotic bacteria from Hippoboscidae (Nováková & Hypša, 2007; Chrudimský et al., 2012; Nováková et al., 2015). Dale et al. (2006) detected a putative obligate endosymbiont from *Pseudolynchia canariensis* which was suggested to represent *Sodalis* bacterium. We detected this symbiont in several members of the Olfersini group and according to our results, it is obligate *Sodalis*-like endosymbiont forming a monophyletic clade, but its congruence with the Olfersini phylogeny is somewhat imperfect (Fig. 3). This incongruence might be a consequence of phylogenetic artefacts likely affecting long branches of *Sodalis* symbionts from *Icosta*. Similar to *Arsenophonus*, *Sodalis* bacteria also establish possible facultative associations, e.g., with *Melophagus ovinus* (Chrudimský et al., 2012; Nováková et al., 2015), *Ornithomya avicularia* (Chrudimský et al., 2012) or *Ornithomya biloba* (this study). *Sodalis* endosymbiont from *Crataerina melbae* was suggested to be obligate (Nováková & Hypša, 2007), but our study did not support this hypothesis since it clusters with free-living *Sodalis praecaptivus*. Interestingly, *Sodalis* endosymbiont of *Microlynchia galapagoensis* was inferred to be closely related to *Sodalis*-like co-symbiont of *Cinara cedri*, which underwent rapid genome deterioration after a replacement of former co-symbiont (Meseguer et al., 2017). These results suggest that there are several loosely associated lineages of *Sodalis* bacteria in louse flies. On one hand, the endosymbiont of *Microlynchia galapagoensis* probably represents a separate (or ancient) *Sodalis* infection, but on the other hand, other *Sodalis* infections seem to be repeatedly acquired from the environment as implied by their relationship to e.g., *Sodalis praecaptivus* (Clayton et al., 2012) (Fig. 3).

Coinfections of obligate and facultative *Arsenophonus* strains in Hippoboscidae (or potentially *Sodalis* in Olfersini) are extremely difficult to recognize using only PCR-acquired 16S rRNA gene. Facultative endosymbionts retain several copies of this gene and thus their 16S rRNA tend to be amplified more likely in PCR than from reduced obligate endosymbionts due to its higher copy number and lower frequency of mutations in primer binding sites. Even though there is a MLST available for *Arsenophonus* bacteria (Duron, Wilkes & Hurst, 2010), it was shown that it is effective only partially (Duron et al., 2014). Since our data are probably also influenced by this setback, we do not speculate which of the detected potentially facultative *Arsenophonus* lineages represent source of ‘ancestors’ for several distinct obligate lineages or which of them were involved in the recent replacement scenario. However, the replacement/independent-origin scenario is well illustrated by endosymbionts from Olfersini (Figs. 2 and 3).

To complement the picture of Hippoboscidae endosymbiosis, we also reconstructed *Wolbachia* evolution. We found three different supergroups: A, B and F (see Table S3). Apparently, there is no coevolution between *Wolbachia* and Hippoboscidae hosts suggesting horizontal transmission between species (Figs. 4A–4B) as common for this bacterium (Schilthuizen & Stouthamer, 1997; Gerth et al., 2014). Since *Wolbachia* seems to be one of the most common donors of genes horizontally transferred to insect genomes, including tsetse flies (Husník et al., 2013; Brelsfoard et al., 2014; Sloan et al., 2014), we cannot rule out that some of *Wolbachia* sequences detected in this study represent HGT insertions into the respective host genomes. The biological role of *Wolbachia* in Hippoboscidae was never examined in spite of its relatively high prevalence in this host group (55%). The F supergroup was detected as the most frequent lineage in Hippoboscidae which is congruent with its common presence in blood-sucking insects such as Streblidae (Morse et al., 2012a), Nycteribiidae (Hosokawa et al., 2012), Amblycera (Covacin & Barker, 2007), and Cimicidae (Hosokawa et al., 2010; Nikoh et al., 2014).

Besides the three main Hippoboscidae symbionts we paid attention to, *Bartonella* spp. that are also widespread among louse flies and bat flies. The infection seems to be fixed only in *Melophagus ovinus* suggesting a mutualistic relationship (Halos et al., 2004), but additional functional data are needed to confirm this hypothesis (Nováková et al., 2015). Nevertheless, deer ked and sheep ked are also suspected of vectoring bartonellosis (Maggi et al., 2009; De Bruin et al., 2015). According to the recent findings, *Bartonella* spp. used to be originally gut symbionts which adapted to pathogenicity (Segers et al., 2016; Neuvonen et al., 2016).

### What is behind dynamics of Hippoboscidae-symbiont associations?

According to our results, symbiosis in the Hippoboscidae group is very dynamic and influenced by frequent symbiont replacements. *Arsenophonus* and *Sodalis* infections seem to be the best resources for endosymbiotic counterparts, but it remains unclear why just these two genera. Both are endowed with several features of free-living/pathogenic bacteria enabling them to enter new host which can be crucial in establishing novel symbiotic association. *Sodalis glossinidius* possesses modified outer membrane protein (OmpA) which is playing an important role in the interaction with the host immune system (Weiss et al., 2008; Weiss, Maltz & Aksoy, 2012). Both *Sodalis* and *Arsenophonus* bacteria retain genes for the type III secretion system (Dale et al., 2001; Wilkes et al., 2010; Chrudimský et al., 2012; Oakeson et al., 2014) allowing pathogenic bacteria to invade eukaryotic cells. Moreover, several strains of these bacteria are cultivable under laboratory conditions (Hypša & Dale, 1997; Dale & Maudlin, 1999; Dale et al., 2006; Darby et al., 2010; Chrudimský et al., 2012; Chari et al., 2015) suggesting that they should be able to survive horizontal transmission. For instance, *Arsenophonus nasoniae* is able to spread by horizontal transfer between species (Duron, Wilkes & Hurst, 2010), while *Sodalis*-allied bacteria have several times successfully replaced ancient symbionts (Conord et al., 2008; Koga et al., 2013; Meseguer et al., 2017).

Whereas the facultative endosymbionts of Hippoboscoidea are widespread in numerous types of tissues such as milk glands, bacteriome, haemolymph, gut, fat body, and reproductive organs (Dale & Maudlin, 1999; Dale et al., 2006; Balmand et al., 2013; Nováková et al., 2015), the obligate endosymbionts are restricted to the bacteriome and milk glands (Aksoy, 1995; Attardo et al., 2008; Balmand et al., 2013; Morse et al., 2013; Nováková et al., 2015). Entering the milk glands ensures vertical transmission of facultative endosymbiont to progeny and better establishment of the infection. Vertical transmission also enables the endosymbiont to hitch-hike with the obligate endosymbiont and because the obligate endosymbiont is inevitably degenerating (Moran, 1996; Wernegreen, 2002), the new co-symbiont can eventually replace it if needed. For instance, *Sodalis melophagi* was shown to appear in both milk glands and bacteriome and to code for the same full set of B-vitamin pathways (including in addition the thiamine pathway) as the obligate endosymbiont *Arsenophonus melophagi* (Nováková et al., 2015). This suggests that it could be potentially capable of shifting from facultative to obligatory lifestyle and replace the *Arsenophonus melophagi* endosymbiont.

We suggest that the complex taxonomic structure of the symbiosis in Hippoboscoidea can be result of multiple replacements, similar to that already suggested for the evolution of symbiosis in *Columbicola* lice (Smith et al., 2013) or mealybugs (Husník & McCutcheon, 2016). Based on the arrangement of the current symbioses in various species of Pupipara, the ancestral endosymbiont was likely either an *Arsenophonus* or *Sodalis* bacterium (given our finding of the potential obligate *Sodalis* lineage in Olfersini). In the course of Pupipara evolution and speciation, this symbiont was repeatedly replaced by different *Arsenophonus* (or *Sodalis* in Olfersini if not ancestral) lineages, as indicated by the lack of phylogenetic congruence and differences in genome reduction, gene order, and GC content in separate *Arsenophonus* lineages (Nováková et al., 2015; Nováková et al., 2016; Figshare: https://figshare.com/s/e1488900c5cb62af69ab). This genomic diversity across the *Arsenophonus* bacteria from distinct Hippoboscidae thus likely reflects their different age correlating with the level of genome reduction in symbiotic bacteria.

## Conclusions

Despite the considerable ecological and geographical variability, the Hippoboscoidea families surprisingly share some aspects of their association with symbiotic bacteria. Particularly, they show high affinity to two bacterial genera, *Arsenophonus* and *Sodalis*. This affinity is not only reflected by frequent occurrence of the bacteria but mainly by their multiple independent acquisitions. Comparisons between the hippoboscid and bacterial phylogenies indicate several independent origins of the symbiosis, although more precise evolutionary reconstruction is still hampered by the uncertainties in hippoboscid phylogenies.

##  Supplemental Information

10.7717/peerj.4099/supp-1 Tables S1–S5 Information about samples, primers, results, and GenBank sequences File includes five tables. Table 1 provides information about samples. Table 2 provides information about Primer names, sequences and products used for PCR amplification and sequencing. Table 3 summarizes results: detected endosymbionts, GC content of their 16S rDNA, and sequences acquired from their host. Table 4 summarizes mitochondrial genes used for phylogeny reconstruction in this study. Table 5 provides accession numbers of GenBank sequences used in this study.

10.7717/peerj.4099/supp-2 Table S6 Information about raw sequences acquired in this study It provides information about raw sequences acquired in this study and their accession numbers.

10.7717/peerj.4099/supp-3 Data S1 Raw sequences acquired in this study File includes raw sequences acquired in this study: symbiont 16S rRNA, *Wolbachia* MLST, host 16S rRNA, host elongation factor, and host cytochrome oxidase subunit I.

10.7717/peerj.4099/supp-4 Figure S1 Phylogeny of Hippoboscoidea based on mitochondrial genes Phylogeny of Hippoboscoidea based on 15 mitochondrial genes. It includes figures of four mitochondrial genomes assembled and annotated in this study which were also used for phylogeny reconstruction.

10.7717/peerj.4099/supp-5 Figure S2 Phylogeny of Hippoboscoidea based on concatenation of 16S rRNA, EF, and COI The phylogeny was reconstructed by BI analysis. Posterior probabilities and bootstrap support are printed upon branches, respectively (asterisk was used for very low or missing bootstrap branch support). Taxa labelled with voucher are newly sequenced in this study. Four main Hippoboscidae groups are colour coded: blue for Glossinidae, light green for Streblidae, dark green for Nycteribiidae, and red for Hippoboscidae.

10.7717/peerj.4099/supp-6 Figure S3 Phylogeny of Hippoboscoidea based on COI The phylogeny was reconstructed by BI analysis. Posterior probabilities and bootstrap support are printed upon branches, respectively (asterisk was used for very low or missing bootstrap branch support). Taxa labelled with voucher are newly sequenced in this study. Genomic COI sequences are labelled with rRNA. Four main Hippoboscidae groups are colour coded: blue for Glossinidae, light green for Streblidae, dark green for Nycteribiidae, and red for Hippoboscidae.

10.7717/peerj.4099/supp-7 Figure S4 Phylogeny of Hippoboscoidea based on EF The phylogeny was reconstructed by BI analysis. Posterior probabilities and bootstrap support are printed upon branches, respectively (asterisk was used for very low or missing bootstrap branch support). Taxa labelled with voucher are newly sequenced in this study. Four main Hippoboscidae groups are colour coded: blue for Glossinidae, light green for Streblidae, dark green for Nycteribiidae, and red for Hippoboscidae.

10.7717/peerj.4099/supp-8 Figure S5 Phylogeny of Hippoboscoidea based on 16S rRNA The phylogeny was reconstructed by BI analysis. Posterior probabilities and bootstrap support are printed upon branches, respectively (asterisk was used for very low or missing bootstrap branch support). Taxa labelled with voucher are newly sequenced in this study. Genomic COI sequences are labelled with rRNA. Four main Hippoboscidae groups are colour coded: blue for Glossinidae, light green for Streblidae, dark green for Nycteribiidae, and red for Hippoboscidae.

10.7717/peerj.4099/supp-9 Figure S6 Phylogeny of Hippoboscidae based on COI The phylogeny was reconstructed by BI analysis. Posterior probabilities and bootstrap support are printed upon branches, respectively (asterisk was used for very low or missing bootstrap branch support). Taxa labelled with voucher are newly sequenced in this study. Genomic COI sequences are labelled with rRNA. Three main families of Hippoboscidae are colour coded: yellow for Lipopteninae (one group), brown for Hippoboscinae (one group), and orange for Ornithomiinae (three groups). Colour squares label branches where are placed main Hippoboscidae groups.

10.7717/peerj.4099/supp-10 Figure S7 Phylogeny of Hippoboscidae based on EF The phylogeny was reconstructed by BI analysis. Posterior probabilities and bootstrap support are printed upon branches, respectively (asterisk was used for very low or missing bootstrap branch support). Taxa labelled with voucher are newly sequenced in this study. Three main families of Hippoboscidae are colour coded: yellow for Lipopteninae (one group), brown for Hippoboscinae (one group), and orange for Ornithomiinae (three groups). Colour squares label branches where are placed main Hippoboscidae groups.

10.7717/peerj.4099/supp-11 Figure S8 Phylogeny of Hippoboscidae based on 16S rRNA The phylogeny was reconstructed by BI analysis. Posterior probabilities and bootstrap support are printed upon branches, respectively (asterisk was used for very low or missing bootstrap branch support). Taxa labelled with voucher are newly sequenced in this study. Three main families of Hippoboscidae are colour coded: yellow for Lipopteninae (one group), brown for Hippoboscinae (one group), and orange for Ornithomiinae (three groups). Colour squares label branches where are placed main Hippoboscidae groups.

10.7717/peerj.4099/supp-12 Figure S9 Phylogeny of *Arsenophonus* within entire Hippoboscoidea based on 16S rRNA Posterior probabilities and bootstrap support are printed upon branches, respectively (asterisk was used for very low or missing bootstrap branch support). Taxa labelled with voucher are newly sequenced in this study. Genomic sequences are labelled with rRNA. Dark blue boxes represent *Arsenophonus* distribution into groups suggested by Morse et al. (2013) and Duron et al. (2014).

10.7717/peerj.4099/supp-13 Figure S10 Phylogeny of all *Arsenophonus* bacteria based on 16S rRNA Posterior probabilities and bootstrap support are printed upon branches, respectively (asterisk was used for very low or missing bootstrap branch support). Taxa labelled with voucher are newly sequenced in this study. Genomic sequences are labelled with rRNA.

## Additional Information and Declarations

### DNA Deposition

The following information was supplied regarding the deposition of DNA sequences: Raw sequences are included in Data S1. Matrices and genomic data of four bacteria used for our analyses are deposited at Figshare (https://figshare.com/s/e1488900c5cb62af69ab).
