# Supplementary material for: Arsenophonus and Sodalis replacements shape evolution of symbiosis in louse flies
Source: PeerJ. 2017 Dec 11;5:e4099. doi: 10.7717/peerj.4099 (PMC5729840; doi:10.7717/peerj.4099)
Supplement: Tables S1–S5 — File includes five tables. Table 1 provides information about samples. Table 2 provides information about Primer names, sequences and products used for PCR amplification and sequencing. Table 3 summarizes results: detected endosymbionts, GC content of their 16S rDNA, and sequences acquired from their host. Table 4 summarizes mitochondrial genes used for phylogeny reconstruction in this study. Table 5 provides accession numbers of GenBank sequences used in this study. [file peerj-05-4099-s001.doc]

**Supplemental table S1:** Samples used in this study characterized by working code, species of louse fly, host species, and locality of sample collection.

| **Voucher** | **Louse fly** | **Host name (family)** | **Locality of sample collection** |
| --- | --- | --- | --- |
|  | **South Africa** |  |  |
| **A2** | *Icosta* sp. 3 | *Accipiter tachiro* (Accipitridae) | Vernon Crookes Nature Reserve, Umzinto, Kwa-Zulu Natal province |
| **G1** | *Icosta* sp. 1 | Gyps fulvus coprotheres (Accipitridae) | Blouberg Nature Reserve, Indermark, Limpopo province |
| **G9** | *Icosta* sp. 1 | Gyps fulvus coprotheres (Accipitridae) | Blouberg Nature Reserve, Indermark, Limpopo province |
| **E1** | *Pseudolynchia* sp. | *Elanus caeruleus* (Accipitridae) | District road north-west of Bosveld Paradys, Limpopo province |
| **CB2** | *Pseudolynchia* sp. | *Calendulauda sabota* (Alaudidae) | De Loskop, Polokwane, Limpopo province |
| **BC1** | *Icosta* sp. 1 | *Columba Guinea* (Columbidae) | Tolwe, Limpopo province |
| **C1** | *Pseudolynchia* sp. | *Columba larvata* (Columbidae) | Vernon Crookes Nature Reserve, Waterfall site, Umzinto, Kwa-Zulu Natal province |
| **CV2** | *Ornithomya biloba* | *Columba livia* (Columbidae) | University of Pretoria, Pretoria, Gauteng province |
| **C4** | *Pseudolynchia* sp. | *Columba livia* (Columbidae) | University of Pretoria, Pretoria, Gauteng province |
| **P8** | *Icosta* sp. 1 | *Pytilia melba* (Estrildidae) | Blouberg Nature Reserve, Indermark, Limpopo province |
| **F5** | *Pseudolynchia* sp. | *Falco amurensis* (Falconidae) | New Castle, Kwa-Zulu Natal province |
| **C2** | *Pseudolynchia* sp. | *Cecropis abyssinica* (Hirundinidae) | Hilltop Farm, Colenso, KwaZulu-Natal province |
| **L3** | *Icosta* sp. 2 | *Lanius collaris* (Laniidae) | Windy Hill, Wartburg, KwaZulu-Natal province |
| **D2** | *Ornithoica* sp. 1 | *Dryoscopus cubla* (Malaconotidae) | Golwe-Vhurivhuri Camp, Venda, Limpopo province |
| **L1** | *Icosta* sp. 2 | *Laniarius ferrugineus* (Malaconotidae) | Happy-Rest Nature Reserve, Limpopo province |
| **L6** | *Icosta* sp. 2 | *Laniarius ferrugineus* (Malaconotidae) | Levubu , Limpopo province |
| **T4** | *Icosta* sp. 2 | *Telophorus zeylonus* (Malaconotidae) | Lambert`s bay, Western Cape province |
| **CL4** | *Icosta* sp. 2 | *Cercotrichas leucophrys* (Muscicapidae) | Blouberg Nature Reserve, Indermark, Limpopo province |
| **CF2** | *Icosta* sp. 2 | *Cossypha caffra* (Muscicapidae) | Happy-Rest Nature Reserve, Limpopo province |
| **CC2** | *Ornithomya avicularia* | *Cossypha dichroa* (Muscicapidae) | Woodbush, Polokwane, Limpopo province |
| **C8** | *Icosta* sp. 2 | *Cossypha natalensis* (Muscicapidae) | University of Limpopo, Limpopo province |
| **BP8** | *Pseudolynchia* sp. | *Passer melanurus* (Passeridae) | Brandvlei, Northern Cape province |
| **E14** | *Pseudolynchia* sp. | *Euplectes orix* (Ploceidae) | Brandvlei, Northern Cape province |
| **P24** | *Pseudolynchia* sp. | *Plocepasser mahali* (Ploceidae) | Polokwane Game Reserve, Polokwane, Limpopo province |
| **P6** | unkown African louse fly | *Ploceus velatus* (Ploceidae) | LC de Villiers sports grounds, Gauteng province |
| **P13** | *Icosta* sp. 2 | *Ploceus velatus* (Ploceidae) | Worcester, Western Cape province |
| **P18** | *Icosta* sp. 1 | *Ploceus velatus* (Ploceidae) | De Loskop, Polokwane, Limpopo province |
| **P16** | *Ornithoica* sp. 1 | *Phyllastrephus terrestris* (Pycnonotidae) | Golwe-Vhurivhuri Camp, Venda, Limpopo province |
| **P20** | *Icosta* sp. 2 | *Phyllastrephus terrestris* (Pycnonotidae) | Blouberg Nature Reserve, Indermark, Limpopo province |
| **C7** | *Icosta* sp. 2 | *Cisticola chiniana* (Cisticolidae) | Polokwane Game Reserve,Polokwane, Limpopo province |
| **C12** | *Icosta* sp. 2 | *Cisticola chiniana* (Cisticolidae) | Blouberg Nature Reserve, Indermark, Limpopo province |
| **P5** | *Icosta* sp. 2 | *Parisoma subcaeruleum* (Sylviidae) | Polokwane Game Reserve, Polokwane, Limpopo province |
| **TB4** | *Pseudolynchia* sp. | *Turdus libonyanus* (Turdidae) | Blouberg Nature Reserve, Indermark, Limpopo province |
| **T1** | *Ornithoica* sp. 1 | *Turdus olivaceus* (Turdidae) | Windy Hill, Wartburg, KwaZulu-Natal Province |
| **Z2** | *Ornithomya avicularia* | *Zoothera gurneyi* (Turdidae) | Woodbush, Polokwane, Limpopo province |
| **Z3** | *Ornithomya avicularia* | *Zoothera gurneyi* (Turdidae) | Woodbush, Polokwane, Limpopo province |
| **U2** | *Pseudolynchia* sp. | *Upupa epops* (Upupidae) | University of Limpopo, Limpopo province |
| **S1** | *Pseudolynchia* sp. | *Streptopelia senegalensis* (Columbidae) | Aloe Forest, Suikerbosrand Nature Reserve, Gauteng province |
| **CB7** | *Icosta* sp. 2 | *Camaroptera brachyura brevicaudata* (Cisticolidae) | Blouberg Nature Reserve, Indermark, Limpopo province |
|  | **Papua New Guinea** |  |  |
| **WAN** | *Icosta* sp. 4 | *Alcedo azurea* (Alcedinidae) | 5°13'36.060"S 145°5'9.000"E |
|  | **Galapagos** |  |  |
| **MG1** | *Microlynchia galapagoensis* | *Zenaida galapagooensis* (Columbidae) | Glapagos |
| **MG2** | *Microlynchia galapagoensis* | *Zenaida galapagooensis* (Columbidae) | Glapagos |
| **MG3** | *Microlynchia galapagoensis* | *Zenaida galapagooensis* (Columbidae) | Glapagos |
| **MG4** | *Microlynchia galapagoensis* | *Zenaida galapagooensis* (Columbidae) | Glapagos |
| **MG6** | *Microlynchia galapagoensis* | *Zenaida galapagooensis* (Columbidae) | Glapagos |
| **MGI1** | *Microlynchia galapagoensis* | *Zenaida galapagooensis* (Columbidae) | Glapagos |
|  | **France** |  |  |
| **CM** | *Crataerina melbae* | *Apus melba* (Apodidae) | Fribany College, Saint-Michel |
|  | **Slovakia** |  |  |
| **HE1** | *Hippobosca equina* | Field sample | Vinné |
|  | **Vietnam** |  |  |
| **I1** | *Ornithoica* sp. 2 | NA | NA |
| **I2** | *Ornithoica* sp. 2 | NA | NA |
| **I3** | *Ornithoica* sp. 3 | NA | NA |
|  | **Czech Republic** |  |  |
| **OA** | *Ornithomya avicularia* | *Upupa epops (*Upupidae) | Bulhary |
| **rRNAob** | *Ornithomya biloba* |  | Pouzdřany |
| **OB** | *Ornithomya biloba* | Hirundo rustica (Hirundinidae) | Nesyt pond |
| **OB2** | *Ornithomya biloba* | Hirundo rustica (Hirundinidae) | Nesyt pond |
| **rRNAcp** | *Crataerina pallida* | *Apus apus* (Apodidae) | Pečky |
| **CP** | *Crataerina pallida* | *Apus apus* (Apodidae) | Pečky |
| **CPA** | *Crataerina pallida* | *Apus apus* (Apodidae) | Pečky |
| **CPB** | *Crataerina pallida* | *Apus apus* (Apodidae) | Pečky |
| **K10c3** | *Lipoptena* sp. | NA | České Budějovice |
| **rRNAam** | *Melophagus ovinus* | *Ovis* sp. (Bovidae) | Krásetín |
| **MO** | *Melophagus ovinus* | *Ovis* sp. (Bovidae) | Krásetín |
| **MO2** | *Melophagus ovinus* | *Ovis* sp. (Bovidae) | Krásetín |
| **rRNAlc** | *Lipoptena cervi* | NA | České Budějovice |
| **JE43** | *Lipoptena cervi* | Field sample | Plav |
| **FH16** | *Lipoptena fortisetosa* | Field sample | Chrášťanský vrch |
| **K13c3** | *Ornithomya avicularia* | NA | NA |
| **OT2** | *Ornithoica turdi* | *Acrocephalus* sp. (Acrocephalidae) | Nesyt pond |
| **K12N** | *Ornithomya fringillina* | Unknown host | Nesyt pond |
| **K14N** | *Ornithoica turdi* | *Acrocephalus scirpaceus* (Acrocephalidae) | Nesyt pond |
| **K15N** | *Ornithoica turdi* | *Emberiza citrinulla* (Emberizidae) | Nesyt pond |
| **K16N** | *Ornithoica turdi* | *Acrocephalus palustris* (Acrocephalidae) | Nesyt pond |
| **K17N** | *Ornithoica turdi* | *Emberiza schoeniclus* (Emberizidae) | Nesyt pond |
| **K18N** | *Ornithomya fringillina* | *Acrocephalus scirpaceus* (Acrocephalidae) | Nesyt pond |
| **K19N** | *Ornithomya avicularia* | *Emberiza citrinulla* (Emberizidae) | Nesyt pond |
| **K20N** | *Ornithomya avicularia* | *Emberiza citrinulla* (Emberizidae) | Nesyt pond |
| **K21N** | Ornithomya avicularia | *Emberiza schoeniclus* (Emberizidae) | Nesyt pond |
| **K22N** | *Ornithoica turdi* | *Acrocephalus scirpaceus* (Acrocephalidae) | Nesyt pond |
| **K23S** | *Ornithomya avicularia* | *Coccothraustes coccothraustes* (Fringillidae) | Nesyt pond |
| **K24N** | *Ornithomya avicularia* | *Parus caeruleus* (Paridae) | Nesyt pond |
| **K25N** | *Ornithomya avicularia* | *Emberiza schoeniclus* (Emberizidae) | Nesyt pond |
| **K26N** | *Ornithomya avicularia* | *Acrocephalus arundinaceus* (Acrocephalidae) | Nesyt pond |
| **K27N** | *Ornithomya fringillina* | *Acrocephalus scirpaceus* (Acrocephalidae) | Nesyt pond |
| **K28N** | *Ornithomya fringillina* | *Acrocephalus scirpaceus* (Acrocephalidae) | Nesyt pond |
| **K29N** | *Ornithomya avicularia* | *Emberiza schoeniclus* (Emberizidae) | Nesyt pond |
| **K30N** | *Ornithomya fringilina* | *Emberiza schoeniclus* (Emberizidae) | Nesyt pond |
| **K31N** | *Ornithomya avicularia* | *Acrocephalus scirpaceus* (Acrocephalidae) | Nesyt pond |
| **K33N** | *Ornithomya biloba* | *Hirudo rustica* (Hirundinidae) | Nesyt pond |
| **K34N** | *Ornithomya avicularia* | *Acrocephalus scirpaceus* (Acrocephalidae) | Nesyt pond |
| **K36N** | *Ornithomya avicularia* | *Acrocephalus scirpaceus* (Acrocephalidae) | Nesyt pond |
| **K37N** | *Ornithomya fringillina* | *Acrocephalus schoenobaenus* (Acrocephalidae) | Nesyt pond |
| **K39N** | *Ornithomya fringillina* | *Acrocephalus scirpaceus* (Acrocephalidae) | Nesyt pond |
| **K40N** | *Ornithomya biloba* | *Carduelis chloris* (Fringillidae) | Nesyt pond |
| **K41N** | *Ornithomya fringillina* | *Emberiza schoeniclus* (Emberizidae) | Nesyt pond |
| **K42N** | *Ornithomya fringillina* | *Acrocephalus scirpaceus* (Acrocephalidae) | Nesyt pond |
| **K43S** | *Ornithomya avicularia* | *Strix aluco* (Strigidae) | Nesyt pond |
| **K44N** | *Ornithomya avicularia* | *Dendrocopos syriacus* (Picidae) | Nesyt pond |
| **Working code** | Bat fly | Host name (family) | Locality of sample collection |
| **NK** | *Nycteribia kolenatii* | *Myotis* sp. (Vespertilionidae) | Lužnice (Czech Repupblic) |

**Supplemental table S2****:** Primer names, sequences and products used for PCR amplification and sequencing.

| **Name** | **Sequence** | **Product (reference)** |
| --- | --- | --- |
| **F40 forward** | GCGGCAAGCCTAACACAT | 16S rDNA (Hypša & Křížek, 2007) |
| **R1060 reverse** | CTTAACCCAACATTTCTCAACACGAG |
| **s16S_eubF forward** | GCTTAACACATGCAAG | 16S rDNA (O'Neill et al., 1992) |
| **16S_eubR reverse** | CCATTGTAGCACGTGT |
| **COIa9 forward** | CCCGGTAAAATTAAAATATAAACTTC | Cytochrome oxidase subunit I (Petersen et al., 2007) |
| **COIb8 reverse** | CCACATTTATTTTGATTTTTTGG |
| **COIa4 forward** | TACAATTTATCGCCTAAACTTCAGCC | Cytochrome oxidase subunit I (Petersen et al., 2007) |
| **CObb12** | TCCAATGCACTAATCTGCCATATTA |
| **A3389lep forward** | TCATAAGTTCARTATCATTG | Cytochrome oxidase subunit I+II (Brown et al., 1994) |
| **S2792lep reverse** | ATACCTCGACGTTATTCAGA |
| **Hip16SF forward** | CGCCTGTTTAACAAAAACAT | 16S mt rDNA (Petersen et al., 2007) |
| **Hip16SR reverse** | TGAACTCAGATCATGTAAGAAA |
| **EF2 forward** | GGAAAT GGGAAAAGGCTCCTTCAAGTA YGCYTGGG | Elongation factor (Moran et al., 1999) |
| **EFS175 reverse** | ATGTGAGCAGTGTGGCAATCCAA |
| **16Swolb99F forward** | TTGTAGCCTGCTATGGTATAACT | 16S rDNA (O’Neill et al., 1992) |
| **16Swolb994R reverse** | GAATAGGTATGATTTTCATGT |  |
| **MLST typing** | | |
| **Name** | Sequence | Product (reference) |
| **gatB_F forward** | GAKTTAAAYCGYGCAGGBGTT | aspartyl/glutamyl-tRNA(Gln) amidotransferase, subunit B (Baldo et al., 2006) |
| **gatB_R reverse** | TGGYAAYTCRGGYAAAGATGA |
| **coxA_F forward** | TTGGRGCRATYAACTTTATAG | cytochrome c oxidase, subunit I (Baldo et al., 2006) |
| **coxA_R reverse** | CTAAAGACTTTKACRCCAGT |
| **hcpA_F forward** | GAAATARCAGTTGCTGCAAA | conserved hypothetical protein (Baldo et al., 2006) |
| **hcpA_R reverse** | GAA AGT YRA GCA AGYTCT G |
| **ftsZ_F forward** | ATYATGGARCATATAAARGATAG | cell division protein (Baldo et al., 2006) |
| **ftsZ_R reverse** | TCRAGYAATGGATTR GATAT |
| **fbpA_F forward** | GCTGCTCCRCTTGGYWTGAT | fructose-bisphosphate aldolase (Baldo et al., 2006) |
| **fbpA_R reverse** | CCRCCAGARAAAAYYACTATTC |

**Supplemental table** **S3****:** Summary of results: detected endosymbionts, GC content of their 16S rDNA, and sequences acquired from their host.

| **Working code** | **16S rDNA host** | **COI** | **EF** | **MT genome** | **16S rDNA symbiont** | **GC (%)** | **16S rDNA *Wolbachia*** | **MLST** |
| --- | --- | --- | --- | --- | --- | --- | --- | --- |
| **A2** |  | A2 | A2 |  | *Sodalis* O | 50.1 | *Wolbachia* A |  |
| **G1** | G1 | G1 | G1 |  |  |  | *Wolbachia* F |  |
| **G9** | G9 | G9 | G9 |  | *Sodalis* O | 49.7 |  |  |
| **E1** | E1 | E1 | E1 |  | *Sodalis* O | 49.5 | *Wolbachia* F |  |
| **CB2** |  |  |  |  | *Arsenophonus* F | 54.1 | *Wolbachia* F |  |
| **BC1** |  |  |  |  | *Arsenophonus* F | 54.7 |  |  |
| **C1** |  |  |  |  | *Arsenophonus* F | 53.9 | *Wolbachia* F |  |
| **CV2** |  | CV2 |  |  | *Sodalis* F | 55.4 | *Wolbachia* B |  |
| **C4** | C4 |  |  |  |  |  | *Wolbachia* F |  |
| **P8** |  |  |  |  |  |  | *Wolbachia* F | *Wolbachia* F |
| **F5** | F5 | F5 | F5 |  | *Arsenophonus* F | 54.0 | *Wolbachia* F | *Wolbachia* F |
| **C2** | C2 | C2 |  |  |  |  | *Wolbachia* F |  |
| **L3** | L3 | L3 |  |  |  |  |  |  |
| **D2** | D2 | D2 |  |  | *Arsenophonus* O | 48.9 |  |  |
| **L1** | L1 | L1 |  |  | *Arsenophonus* F | 53.8 | *Wolbachia* B |  |
| **L6** |  | L6 |  |  | *Arsenophonus* F | 55.1 | *Wolbachia* F | *Wolbachia* F |
| **T4** | T4 |  | T4 |  | *Arsenophonus* F | 53.9 |  |  |
| **CL4** |  |  | CL4 |  |  |  |  |  |
| **CF2** | CF2 | CF2 | CF2 |  |  |  | *Wolbachia* F |  |
| **CC2** |  |  | CC2 |  |  |  |  |  |
| **C8** |  |  | C8 |  | *Sodalis* O | 49.7 |  |  |
| **BP8** |  |  |  |  | *Arsenophonus* F/O | 52.6 |  |  |
| **E14** |  |  |  |  | *Arsenophonus* F/O | 52.6 | *Wolbachia* F |  |
| **P24** | P24 | P24 | P24 |  | *Arsenophonus* F | 53.1 | *Wolbachia* F |  |
| **P6** |  | P6 |  |  | *Arsenophonus* F |  | *Wolbachia* F |  |
| **P13** | P13 |  | P13 |  | *Sodalis* O | 50.5 |  |  |
| **P18** |  |  | P18 |  |  |  |  |  |
| **P16** | P16 | P16 |  |  | *Arsenophonus* O | 49.3 |  |  |
| **P20** |  |  | P20 |  | *Arsenophonus* F | 53.7 | *Wolbachia* B | *Wolbachia* B |
| **C7** |  | C7 |  |  |  |  | *Wolbachia* B |  |
| **C12** |  |  | C12 |  |  |  |  |  |
| **P5** | P5 | P5 | P5 |  | *Sodalis* O | 50.3 | *Wolbachia* B |  |
| **TB4** |  |  | TB4 |  |  |  |  |  |
| **T1** |  | T1 |  |  | *Arsenophonus* O | 48.9 |  |  |
| **Z2** | Z2 | Z2 |  |  |  |  | *Wolbachia* B |  |
| **Z3** | Z3 | Z3 | Z3 |  | *Arsenophonus* O | 51 |  |  |
| **U2** | U2 | U2 | U2 |  | *Sodalis* O | 47.8 |  |  |
| **S1** | S1 |  |  |  |  |  | *Wolbachia* F |  |
| **CB7** |  |  | CB7 |  |  |  |  |  |
| **WAN** | WAN |  | WAN |  | *Sodalis O* | 51.1 | *Wolbachia* A | *Wolbachia* A |
| **MG1** | MG1 | MG1 |  |  | *Sodalis* F | 55.5 | *Wolbachia* B |  |
| **MG2** |  |  | MG2 |  | *Sodalis* F | 55.5 |  |  |
| **MG3** |  |  |  |  | *Sodalis* F | 55.5 |  |  |
| **MG4** |  |  |  |  | *Sodalis* F | 55.5 |  |  |
| **MG6** |  |  |  |  |  |  |  | *Wolbachia* B |
| **MGI1** |  |  | MGI1 |  |  |  |  |  |
| **CM** | CM |  |  |  |  |  |  |  |
| **HE1** |  |  | HE1 |  |  |  |  |  |
| **OA** | OA | OA | OA |  | *Arsenophonus* O | 50.2 |  |  |
| **rRNAob** |  | rRNAob |  | rRNAob | *Arsenophonus* O, *Sodalis* F | 49.4, 55.1 | *Wolbachia* A | *Wolbachia* A |
| **OB** | OB | OB | OB |  | *Arsenophonus* F | 54.1 |  |  |
| **OB2** |  |  | OB2 |  |  |  |  |  |
| **rRNAcp** |  | rRNAcp |  | rRNAcp | *Arsenophonus* F/O | 53.4 |  |  |
| **CP** |  | CP | CP |  |  |  | *Wolbachia* A | *Wolbachia* A |
| **CPA** | CPA |  | CPA |  |  |  |  |  |
| **CPB** | CPB |  | CPB |  |  |  |  |  |
| **rRNAam** |  | rRNAam |  | rRNAam | *Arsenophonus* O | 52.5 |  |  |
| **MO** | MO |  | MO |  |  |  | *Wolbachia* F | *Wolbachia* F |
| **MO2** | MO2 |  | MO2 |  |  |  |  |  |
| **K10c3** |  |  |  |  | *Arsenophonus* O | 50.5 |  |  |
| **rRNAlc** |  |  |  | rRNAlc | *Arsenophonus* O | 50.6 |  |  |
| **JE43** | JE43 | JE43 | JE43 |  |  |  | *Wolbachia* F |  |
| **FH16** | FH16 | FH16 | FH16 |  |  |  | *Wolbachia* B |  |
| **OT2** | OT2 |  | OT2 |  | *Arsenophonus* O | 42.8 | *Wolbachia* F |  |
| **K13c3** |  |  |  |  | *Arsenophonus* F | 54.1 |  |  |
| **K12N** |  | K12N |  |  |  |  |  |  |
| **K14N** |  | K14N | K14N |  |  |  |  |  |
| **K15N** |  | K15N | K15N |  | *Arsenophonus* O | 49.2 |  |  |
| **K16N** |  | K16N | K16N |  | *Arsenophonus* O | 49.5 |  |  |
| **K17N** |  | K17N | K17N |  |  |  |  |  |
| **K18N** |  | K18N | K18N |  |  |  |  |  |
| **K19N** |  | K19N |  |  |  |  |  |  |
| **K20N** |  | K20N |  |  |  |  |  |  |
| **K21N** |  | K21N |  |  |  |  |  |  |
| **K22N** |  | K22N |  |  | *Arsenophonus* O | 49.3 |  |  |
| **K23N** |  | K23N |  |  |  |  |  |  |
| **K24N** |  | K24N | K24N |  | *Arsenophonus* O | 49.9 |  |  |
| **K25N** |  | K25N | K25N |  | *Arsenophonus* O | 50.6 |  |  |
| **K26N** |  | K26N | K26N |  | *Arsenophonus* O | 50.5 |  |  |
| **K27N** |  | K27N | K27N |  |  |  |  |  |
| **K28N** |  | K28N |  |  |  |  |  |  |
| **K29N** |  | K29N | K29N |  |  |  |  |  |
| **K30N** |  | K30N | K30N |  |  |  |  |  |
| **K31N** |  | K31N |  |  |  |  |  |  |
| **K33N** |  | K33N |  |  |  |  |  |  |
| **K34N** |  | K34N |  |  |  |  |  |  |
| **K36N** |  | K36N |  |  |  |  |  |  |
| **K37N** |  | K37N |  |  |  |  |  |  |
| **K39N** |  | K39N |  |  |  |  |  |  |
| **K40N** |  | K40N | K40N |  |  |  |  |  |
| **K41N** |  | K41N |  |  |  |  |  |  |
| **K42N** |  | K42N |  |  |  |  |  |  |
| **K43S** |  | K43S |  |  |  |  |  |  |
| **K44N** |  | K44N |  |  |  |  |  |  |
| **NK** |  |  | NK |  | *Arsenophonus* O | 47.7 |  | *Wolbachia* F |

**Supplemental table S4:** Summary of mitochondrial genes used for phylogeny reconstruction in this study.

| **Abbreviation** | **Gene** |
| --- | --- |
| **COI** | Cytochrome oxidase subunit 1 |
| **COII** | Cytochrome oxidase subunit 2 |
| **COII** | Cytochrome oxidase subunit 3 |
| **ATP6** | ATP synthase subunit 6 |
| **ATP8** | ATP synthase subunit 8 |
| **NADH1** | NADH dehydrogenase subunit 1 |
| **NADH2** | NADH dehydrogenase subunit 2 |
| **NADH3** | NADH dehydrogenase subunit 3 |
| **NADH4** | NADH dehydrogenase subunit 4 |
| **NADH4L** | NADH dehydrogenase subunit 4L |
| **NADH5** | NADH dehydrogenase subunit 5 |
| **NADH6** | NADH dehydrogenase subunit 6 |
| **CYTB** | Cytochrome B |
| **16S** | rRNA large subunit |
| **12S** | rRNA small subunit |

**Supplemental table S5:** Accession numbers of GenBank sequences used in this study.

| **Accession number** | | **Name of sequence** | | |
| --- | --- | --- | --- | --- |
| ***Arsenophonus*** | | | | |
| **FJ265819** | Arsenophonus symbiont of Melophagus ovinus 16S ribosomal RNA gene | | | |
| **DQ115536** | Candidatus Arsenophonus arthropodicus 16S ribosomal RNA gene | | | |
| **FJ265793** | Arsenophonus symbiont of Hippobosca camelina clone 1 16S ribosomal RNA gene | | | |
| **FJ265816** | Arsenophonus symbiont of Ornithomya biloba clone 1 16S ribosomal RNA gene | | | |
| **KC597727** | Bacterium endosymbiont of Leptocyclopodia n. sp. 1 clone JAE1033 16S ribosomal RNA gene | | | |
| **KC597759** | Bacterium endosymbiont of Megastrebla sp. clone P4511.2 16S ribosomal RNA gene | | | |
| **KC597760** | Bacterium endosymbiont of Brachytarsina sp. 1 clone P4635 16S ribosomal RNA gene | | | |
| **FJ265803** | Arsenophonus symbiont of Nycteribia sp. 16S ribosomal RNA gene | | | |
| **KC597714** | Bacterium endosymbiont of Basilia tiptoni clone CW26.10.03-3 16S ribosomal RNA gene | | | |
| **KC597734** | Bacterium endosymbiont of Basilia boardmani clone JAW05 16S ribosomal RNA gene | | | |
| **EU727111** | Arsenophonus symbiont of Hippobosca equina 16S ribosomal RNA gene | | | |
| **KC597770** | Bacterium endosymbiont of Dipseliopoda biannulata clone VR35 16S ribosomal RNA gene | | | |
| **U91786** | Arsenophonus triatominarum 16S ribosomal RNA gene | | | |
| **KC597737** | Bacterium endosymbiont of Ornithomyia fringillina clone MCP-05-165 16S ribosomal RNA gene | | | |
| **M90801** | Arsenophonus nasoniae 16S ribosomal RNA | | | |
| **KC597757** | Bacterium endosymbiont of Leptocyclopodia ferrarii ssp. 1 clone P2650 16S ribosomal RNA gene | | | |
| **KC597762** | Bacterium endosymbiont of Anatrichobius scorzai clone PMV618 16S ribosomal RNA gene | | | |
| **KC597756** | Bacterium endosymbiont of Leptocyclopodia brevicula clone P2466 16S ribosomal RNA gene | | | |
| **KC597710** | Bacterium endosymbiont of Eucampsipoda africana clone BDP4416 16S ribosomal RNA gene | | | |
| **KC597754** | Bacterium endosymbiont of Eucampsipoda cf. Latisterna clone P636 16S ribosomal RNA gene | | | |
| **KC597758** | Bacterium endosymbiont of Eucampsipoda inermis clone P4496 16S ribosomal RNA gene | | | |
| **KC597716** | Bacterium endosymbiont of Lipoptena cervi clone DK 16S ribosomal RNA gene | | | |
| **KC597771** | Bacterium endosymbiont of Lipoptena depressa clone WSPA2000 16S ribosomal RNA gene | | | |
| **FJ265794** | Arsenophonus symbiont of Lipoptena cervi clone 1 16S ribosomal RNA gene | | | |
| **FJ265798** | Arsenophonus symbiont of Lipoptena fortisetosa clone 2 16S ribosomal RNA gene | | | |
| **FJ265799** | Arsenophonus symbiont of Lipoptena sp. 16S ribosomal RNA gene | | | |
| **J01874** | Proteus vulgaris 16S ribosomal RNA | | | |
| **AB626123** | Proteus mirabilis gene for 16S rRNA | | | |
| **FJ265787** | Arsenophonus symbiont of Ornithomya chloropus 16S ribosomal RNA gene | | | |
| **FJ265815** | Arsenophonus symbiont of Ornithomya avicularia clone 1 16S ribosomal RNA gene | | | |
| **KC597718** | Bacterium endosymbiont of Paradyschiria fusca clone DR05088 16S ribosomal RNA gene | | | |
| **DQ314769** | Arsenophonus endosymbiont of Trichobius caecus clone 2 16S ribosomal RNA gene | | | |
| **DQ314773** | Arsenophonus endosymbiont of Trichobius longipes clone 15 16S ribosomal RNA gene | | | |
| **KC597717** | Bacterium endosymbiont of Trichobius intermedius clone DR05076 16S ribosomal RNA gene | | | |
| **KC597768** | Bacterium endosymbiont of Aspidoptera falcata clone TK135285 16S ribosomal RNA gene | | | |
| **FJ265818** | Arsenophonus symbiont of Trichobius sp. 16S ribosomal RNA gene | | | |
| **DQ314775** | Arsenophonus endosymbiont of Trichobius parasiticus clone 10 16S ribosomal RNA gene | | | |
| **KC597739** | Bacterium endosymbiont of Trichobius parasiticus clone MEX05 16S ribosomal RNA gene | | | |
| **KC597769** | Bacterium endosymbiont of Aspidoptera phyllostomatis clone TK135293 16S ribosomal RNA gene | | | |
| **KC597719** | Bacterium endosymbiont of Trichobius neotropicus clone DR05139.3 16S ribosomal RNA gene | | | |
| **KC597748** | Bacterium endosymbiont of Trichobius dugesioides clone MN144 16S ribosomal RNA gene | | | |
| **JN561339** | Arsenophonus endosymbiont of Trichobius major strain BF104 16S ribosomal RNA gene | | | |
| **KC597735** | Bacterium endosymbiont of Trichobius cf. Cernyi clone KD0802111.1 16S ribosomal RNA gene | | | |
| **KC597711** | Bacterium endosymbiont of Trichobius frequens clone BFPaperpupa1 16S ribosomal RNA gene | | | |
| **KC597721** | Bacterium endosymbiont of Megistopoda aranea clone EHA49-M 16S ribosomal RNA gene | | | |
| **KC597724** | Bacterium endosymbiont of Paratrichobius longicrus clone FG10 16S ribosomal RNA gene | | | |
| **KC597744** | Bacterium endosymbiont of Strebla diphyllae clone MEX15.1 16S ribosomal RNA gene | | | |
| **FJ265804** | Arsenophonus symbiont of Nycteribia kolenatii clone 1 16S ribosomal RNA gene | | | |
| **KC597718** | Bacterium endosymbiont of Paradyschiria fusca clone DR05088 16S ribosomal RNA gene | | | |
| **DQ314769** | Arsenophonus endosymbiont of Trichobius caecus clone 2 16S ribosomal RNA gene | | | |
| **DQ314773** | Arsenophonus endosymbiont of Trichobius longipes clone 15 16S ribosomal RNA gene | | | |
| **KC597717** | Bacterium endosymbiont of Trichobius intermedius clone DR05076 16S ribosomal RNA gene | | | |
| **KC597768** | Bacterium endosymbiont of Aspidoptera falcata clone TK135285 16S ribosomal RNA gene | | | |
| **FJ265818** | Arsenophonus symbiont of Trichobius sp. 16S ribosomal RNA gene | | | |
| **DQ314775** | Arsenophonus endosymbiont of Trichobius parasiticus clone 10 16S ribosomal RNA gene | | | |
| **KC597739** | Bacterium endosymbiont of Trichobius parasiticus clone MEX05 16S ribosomal RNA gene | | | |
| **KC597769** | Bacterium endosymbiont of Aspidoptera phyllostomatis clone TK135293 16S ribosomal RNA gene | | | |
| **KC597719** | Bacterium endosymbiont of Trichobius neotropicus clone DR05139.3 16S ribosomal RNA gene | | | |
| **KC597748** | Bacterium endosymbiont of Trichobius dugesioides clone MN144 16S ribosomal RNA gene | | | |
| **JN561339** | Arsenophonus endosymbiont of Trichobius major strain BF104 16S ribosomal RNA gene | | | |
| **KC597735** | Bacterium endosymbiont of Trichobius cf. cernyi clone KD0802111.1 16S ribosomal RNA gene | | | |
| **KC597711** | Bacterium endosymbiont of Trichobius frequens clone BFPaperpupa1 16S ribosomal RNA gene | | | |
| **KC597721** | Bacterium endosymbiont of Megistopoda aranea clone EHA49-M 16S ribosomal RNA gene | | | |
| **KC597724** | Bacterium endosymbiont of Paratrichobius longicrus clone FG10 16S ribosomal RNA gene | | | |
| **KC597744** | Bacterium endosymbiont of Strebla diphyllae clone MEX15.1 16S ribosomal RNA gene | | | |
| **FJ265804** | Arsenophonus symbiont of Nycteribia kolenatii clone 1 16S ribosomal RNA gene | | | |
| **AB632412** | Bacterium endosymbiont of Nycteribia allotopa gene for 16S ribosomal RNA isolate NyAl1 | | | |
| **AB632427** | Bacterium endosymbiont of Nycteribia pleuralis gene for 16S ribosomal RNA isolate NyPl1 | | | |
| **AB632423** | Bacterium endosymbiont of Nycteribia pygmaea gene for 16S ribosomal RNA isolate NyPy1 | | | |
| **KC597728** | Bacterium endosymbiont of Phthiridium cf. tonkinensis clone JAE1224 16S ribosomal RNA gene | | | |
| **KC597736** | Bacterium endosymbiont of Phthiridium sp. clone MALA15 16S ribosomal RNA gene | | | |
| **KC597713** | Bacterium endosymbiont of Penicillidia dufourii ssp. clone CHI05 16S ribosomal RNA gene | | | |
| **AB632404** | Bacterium endosymbiont of Basilia truncata gene for 16S ribosomal RNA isolate BaTr1 | | | |
| **KC597772** | Bacterium endosymbiont of Basilia nattereri clone ZAG03 16S ribosomal RNA gene | | | |
| **KC597752** | Bacterium endosymbiont of Phthiridium chinense clone P440 16S ribosomal RNA gene | | | |
| **FJ265805** | Arsenophonus symbiont of Penicillidia sp. 16S ribosomal RNA gene | | | |
| **FJ265817** | Arsenophonus symbiont of Penicillidia monoceros 16S ribosomal RNA gene | | | |
| **KC597726** | Bacterium endosymbiont of Penicillidia oceanica acuminata clone JAE978 16S ribosomal RNA gene | | | |
| **KC597746** | Bacterium endosymbiont of Trichobius sp. clone MEX2009E3 16S ribosomal RNA gene | | | |
| **EF467859** | Photorhabdus temperata subsp. stackebrandtii strain GPS11 16S ribosomal RNA gene | | | |
| **AY660027** | Xenorhabdus bovienii 16S ribosomal RNA gene | | | |
| **HM345970** | Providencia sp. 16S ribosomal RNA gene | | | |
| **JX853024** | Bacterium endosymbiont of Nycterophylia coxata clone DR05037 16S ribosomal RNA gene | | | |
| **JX853062** | Bacterium endosymbiont of Nycterophylia parnelli clone MEX2009A4.1 16S ribosomal RNA gene | | | |
| **JX853027** | Bacterium endosymbiont of Nycterophylia n. sp. clone DR05084 16S ribosomal RNA gene | | | |
| **KC597723** | Bacterium endosymbiont of Trichobius cf. yunkeri clone FG08 16S ribosomal RNA gene | | | |
| **KC597745** | Bacterium endosymbiont of Trichobius n. sp. clone MEX17 16S ribosomal RNA gene | | | |
| **U91515** | Phlomobacter fragariae 16S rRNA gene | | | |
| **EF647590** | Candidatus Riesia pediculicola 16S small subunit ribosomal RNA gene | | | |
| **AB632437** | Bacterium endosymbiont of Penicillidia jenynsii gene for 16S ribosomal RNA isolate PeJe6 | | | |
| **AB632411** | Bacterium endosymbiont of Basilia rybini gene for 16S ribosomal RNA isolate BaRy1 | | | |
| **FJ265814** | Arsenophonus symbiont of Ornithomya avicularia clone 2 16S ribosomal RNA gene | | | |
| **ftp://ftp.ncbi.nih.gov/genomes/genbank/bacteria/Arsenophonus_endosymbiont_of_Nilaparvata_lugens/latest_assembly_versions/GCA_000757905.1_ASM75790v1** | | | | Arsenophonus nilaparvatae |
| **FJ265811** | Arsenophonus symbiont of Ornithomya avicularia clone 3 16S ribosomal RNA gene | | | |
| **DQ314778** | Arsenophonus endosymbiont of Lipoptena cervi clone 3 16S ribosomal RNA gene | | | |
| **FJ265813** | Arsenophonus symbiont of Ornithomya biloba clone 2 16S ribosomal RNA gene | | | |
| **EF110573** | Candidatus Riesia pediculischaeffi 4.30.2003.8 16S ribosomal RNA gene | | | |
| **FJ265802** | Arsenophonus symbiont of Nycteribia kolenatii clone 2 16S ribosomal RNA gene | | | |
| **KC597761** | Bacterium endosymbiont of Brachytarsina sp. 2 clone P4643 16S ribosomal RNA gene | | | |
| **EF110574** | Candidatus Riesia pthiripubis 8.14.2002.1 16S ribosomal RNA gene | | | |
| **HM594692** | Arsenophonus endosymbiont of Trialeurodes vaporariorum isolate trial16S 16S ribosomal RNA gene | | | |
| **KP183240** | Arsenophonus endosymbiont of Colletes halophilus isolate CH3 16S ribosomal RNA gene | | | |
| **KP183241** | Arsenophonus endosymbiont of Colletes cunicularius isolate CCu1 16S ribosomal RNA gene | | | |
| **KP183242** | Arsenophonus endosymbiont of Colletes hederae isolate CoH1 16S ribosomal RNA gene | | | |
| **AB038366** | Arsenophonus endosymbiont of Diaphorina citri gene for 16S rRNA | | | |
| **JN990929** | Arsenophonus endosymbiont of Ericerus pela clone 9 16S ribosomal RNA gene | | | |
| **FJ265801** | Arsenophonus symbiont of Myzocallis sp. clone 1 16S ribosomal RNA gene | | | |
| **AY264665** | Arsenophonus endosymbiont of Aleuroplatus gelatinosus 16S ribosomal RNA gene | | | |
| **HM594694** | Arsenophonus endosymbiont of Eupelmus vesicularis isolate evesrom16S 16S ribosomal RNA gene | | | |
| **EU727114** | Arsenophonus symbiont of Pyrrhocoris apterus 16S ribosomal RNA gene | | | |
| **HM594697** | Arsenophonus endosymbiont of Spalangia endius isolate sendius16S 16S ribosomal RNA gene | | | |
| **AB772262** | Arsenophonus endosymbiont of Philaenarcys bilineata gene for 16S ribosomal RNA | | | |
| **AY264663** | Arsenophonus endosymbiont of Acanthaleyrodes styraci 16S ribosomal RNA gene | | | |
| **AY264673** | Arsenophonus endosymbiont of Australiococcus greville 16S ribosomal RNA gene | | | |
| **AY264668** | Arsenophonus endosymbiont of Neomaskellia andropogonis 16S ribosomal RNA gene | | | |
| **AY264667** | Arsenophonus endosymbiont of Dialeurodes hongkongensis 16S ribosomal RNA gene | | | |
| **AY264666** | Arsenophonus endosymbiont of Aleyrodes elevatus 16S ribosomal RNA gene | | | |
| **FJ655543** | Arsenophonus symbiont of Stomaphis quercus 16S ribosomal RNA gene | | | |
| **AB823665** | Arsenophonus endosymbiont of Aphis craccivora gene for 16S ribosomal RNA | | | |
| **FJ655542** | Arsenophonus symbiont of Stomaphis longirostris 16S ribosomal RNA gene | | | |
| **LN890583** | Arsenophonus endosymbiont of Apis mellifera partial 16S rRNA gene | | | |
| **AY264672** | Arsenophonus endosymbiont of Trialeurodes vaporariorum 16S ribosomal RNA | | | |
| **AY587142** | Arsenophonus endosymbiont of Aleurodicus dugesii 16S ribosomal RNA gene | | | |
| **DQ508169** | Arsenophonus endosymbiont of Triatoma rubrofasciata clone 2E 16S ribosomal RNA gene | | | |
| **LN890584** | Arsenophonus endosymbiont of Apis dorsata partial 16S rRNA gene | | | |
| **LN890585** | Arsenophonus endosymbiont of Apis florea partial 16S rRNA gene | | | |
| **DQ517447** | Arsenophonus endosymbiont of Meccus mazzottii 16S ribosomal RNA gene | | | |
| **AY587140** | Arsenophonus endosymbiont of Trialeurodes hutchingsi 16S ribosomal RNA gene | | | |
| **HM594695** | Arsenophonus endosymbiont of Spalangia cameroni isolate scamrus16S 16S ribosomal RNA gene | | | |
| **LN829899** | Arsenophonus endosymbiont of Bemisia tabaci partial 16S rRNA gene | | | |
| **DQ508198** | Arsenophonus endosymbiont of Eratyrus mucronatus 16S ribosomal RNA gene | | | |
| **AY264669** | Arsenophonus endosymbiont of Siphoninus phillyreae 16S ribosomal RNA gene | | | |
| **EU727115** | Arsenophonus symbiont of Protocalliphora sp. 16S ribosomal RNA gene | | | |
| **AY587141** | Arsenophonus endosymbiont of Aleyrodes proletella 16S ribosomal RNA gene | | | |
| **FJ265790** | Arsenophonus symbiont of Technomyrmex albipes clone 2 16S ribosomal RNA gene | | | |
| **KM593930** | Arsenophonus endosymbiont of Aphis gossypii clone Nanjing cotton 16S ribosomal RNA gene | | | |
| **KR337979** | Arsenophonus symbiont of Macrosteles laevis clone MlA21 16S ribosomal RNA gene | | | |
| **AY264664** | Arsenophonus endosymbiont of Aleurodicus dispersus 16S ribosomal RNA gene | | | |
| **FJ655541** | Arsenophonus symbiont of Stomaphis takahashii 16S ribosomal RNA gene | | | |
| **FJ655540** | Arsenophonus symbiont of Stomaphis fagi 16S ribosomal RNA gene | | | |
| **AY264671** | Arsenophonus endosymbiont of Tetraleurodes mori 16S ribosomal RNA gene | | | |
| **FJ265788** | Arsenophonus symbiont of Aenictus huonicus 16S ribosomal RNA gene | | | |
| **AY265348** | Arsenophonus endosymbiont of Dermacentor variabilis isolate 17.10.2 16S ribosomal RNA gene | | | |
| **KX146212** | Uncultured bacterium clone Lnlae630093 16S ribosomal RNA gene | | | |
| **KX146214** | Uncultured bacterium clone Nescp630095 16S ribosomal RNA gene | | | |
| **LN878131** | Candidatus Providencia siddallii partial 16S rRNA gene | | | |
| **KY606569** | Proteus hauseri strain DSM 14437 16S ribosomal RNA gene | | | |
| **KY684830** | Proteus terrae strain BR3 16S ribosomal RNA gene | | | |
| **KY885156** | Proteus penneri strain KAU-15 16S ribosomal RNA gene | | | |
| **KY311817** | Xenorhabdus stockiae isolate CS37 16S ribosomal RNA gene | | | |
| **D78010** | Xenorhabdus poinarii gene for 16S rRNA | | | |
| **X82253** | X.poinarii 16S rRNA gene | | | |
| **NR114965** | Xenorhabdus indica strain 28 16S ribosomal RNA gene | | | |
| **X82254** | X.beddingii 16S rRNA gene | | | |
| **D78008** | Xenorhabdus japonica gene for 16S rRNA | | | |
| **KY863498** | Providencia stuartii strain Bp-40 16S ribosomal RNA gene | | | |
| **KY027172** | Providencia vermicola strain AMJ240 16S ribosomal RNA gene | | | |
| **KY967319** | Providencia rettgeri strain PR 01 16S ribosomal RNA gene | | | |
| **KY606570** | Providencia heimbachae strain NCTC12003 16S ribosomal RNA gene | | | |
| **KY606582** | Providencia burhodogranariea DSM 19968 strain B 16S ribosomal RNA gene, | | | |
| **X82249** | Photorhabdus temperata 16S rRNA gene | | | |
| **X82248** | Photorhabdus luminescens subsp. luminescens 16S rRNA gene | | | |
| **KY316165** | Xenorhabdus nematophila strain FUM1 16S ribosomal RNA gene | | | |
| ***Sodalis*** | | | | |
| **DQ115535** | Primary endosymbiont of Pseudolynchia canariensis 16S ribosomal RNA gene | | | |
| **AB507712** | Secondary endosymbiont of Curculio sikkimensis gene for 16S ribosomal RNA | | | |
| **AB604872** | Sodalis secondary endosymbiont of Archarius roelofsi gene for 16S ribosomal RNA | | | |
| **AB604873** | Sodalis secondary endosymbiont of Curculio hachijoensis gene for 16S ribosomal RNA | | | |
| **JN872639** | Bacterium endosymbiont of Ornithomya avicularia 16S ribosomal RNA gene | | | |
| **EF174495** | Sodalis endosymbiont of Craterina melbae strain CMS06 16S ribosomal RNA gene | | | |
| **JN872637** | Candidatus Sodalis melophagi strain CZT 16S ribosomal RNA gene | | | |
| **M99060** | Glossina pallidipes S-endosymbiont (tsetse) endosymbiont ribosomal RNA small subunit | | | |
| **U64869** | Glossina austeni S-endosymbiont 16S ribosomal RNA gene | | | |
| **U64867** | Glossina palpalis S-endosymbiont 16S ribosomal RNA gene | | | |
| **NR074525** | Sodalis glossinidius str. 'morsitans' strain morsitans 16S ribosomal RNA | | | |
| **U64868** | Glossina fuscipes S-endosymbiont 16S ribosomal RNA gene | | | |
| **U64870** | Glossina brevipalpis S-endosymbiont 16S ribosomal RNA gene | | | |
| **AM946408** | Uncultured bacterium partial 16S rRNA gene, isolated from Tetropium castaneum | | | |
| **JN872638** | Bacterium endosymbiont of Rhamphus pulicarius 16S ribosomal RNA gene | | | |
| **AY126638** | Primary endosymbiont of Sitophilus granarius 16S ribosomal RNA gene | | | |
| **AM774412** | Biostraticola tofi partial 16S rRNA gene, type strain BF36T | | | |
| **AF005235** | Sitophilus oryzae principal endosymbiont 16S ribosomal RNA gene | | | |
| **JQ063439** | Endosymbiont of Columbicola columbae strain 07.15.02 16S ribosomal RNA gene | | | |
| **HM196339** | Brenneria quercina strain W3L22 16S ribosomal RNA gene | | | |
| **HM585373** | Serratia rubidaea strain E9 16S ribosomal RNA gene | | | |
| **DQ133565** | Uncultured proteobacterium from Puto sp. 16S ribosomal RNA gene | | | |
| **M85269** | Endosymbiont Sitophilus zeamais 16S ribosomal RNA | | | |
| **J01874.** | Proteus vulgaris 16S ribosomal RNA | | | |
| **AB626123** | Proteus mirabilis gene for 16S rRNA, partial sequence | | | |
| **EF467859** | Photorhabdus temperata subsp. stackebrandtii strain GPS11 16S ribosomal RNA gene | | | |
| **AY660027** | Xenorhabdus bovienii 16S ribosomal RNA gene | | | |
| **AB809072** | Sodalis-like secondary symbiont of Antestiopsis thunbergii gene for 16S ribosomal RNA | | | |
| **AB915773** | Sodalis endosymbiont of Dolycoris baccarum gene for 16S ribosomal RNA | | | |
| **AB915770** | Sodalis endosymbiont of Elasmucha putoni gene for 16S ribosomal RNA | | | |
| **AB915780** | Sodalis endosymbiont of Picromerus lewisi gene for 16S ribosomal RNA | | | |
| **AB915775** | Sodalis endosymbiont of Lelia decempunctata gene for 16S ribosomal RNA | | | |
| **AB915779** | Sodalis endosymbiont of Palomena angulosa gene for 16S ribosomal RNA | | | |
| **AB915774** | Sodalis endosymbiont of Glaucias subpunctatus gene for 16S ribosomal RNA | | | |
| **AB915776** | Sodalis endosymbiont of Nezara antennata gene for 16S ribosomal RNA | | | |
| **AB821283** | Uncultured bacterium gene for 16S ribosomal RNA, clone: kaiwa2013 kunu7 | | | |
| **AB821278** | Uncultured bacterium gene for 16S ribosomal RNA, clone: kaiwa2013 kunu2 | | | |
| **AB821289** | Uncultured bacterium gene for 16S ribosomal RNA, clone: kaiwa2013 kunu13 | | | |
| **AB915771** | Sodalis endosymbiont of Aelia fieberi gene for 16S ribosomal RNA | | | |
| **AB915781** | Sodalis endosymbiont of Piezodorus hybneri gene for 16S ribosomal RNA | | | |
| **AB915783** | Sodalis endosymbiont of Rhopalus sapporensis gene for 16S ribosomal RNA | | | |
| **AB915782** | Sodalis endosymbiont of Poecilocoris lewisi gene for 16S ribosomal RNA | | | |
| **AB571330** | Uncultured bacterium gene for 16S rRNA, clone: NK-2010-sd-01 | | | |
| **AB772196** | Sodalis-like endosymbiont of Philaenus spumarius gene for 16S ribosomal RNA | | | |
| **KX146202** | Uncultured bacterium clone Axsp714095.1 16S ribosomal RNA gene | | | |
| **KX146199** | Uncultured bacterium clone Echin317092 16S ribosomal RNA gene | | | |
| **KX146215.1** | Uncultured bacterium clone Sathrax426093 16S ribosomal RNA gene | | | |
| ***Wolbachia*** | | | | |
| **DQ115537** | Wolbachia endosymbiont of Pseudolynchia canariensis Type 1 16S ribosomal RNA gene | | | |
| **AY316361** | Wolbachia endosymbiont of Cimex lectularius 16S ribosomal RNA gene | | | |
| **KC677586** | Wolbachia endosymbiont of Tribolium confusum 16S ribosomal RNA gene | | | |
| **DQ115538** | Wolbachia endosymbiont of Pseudolynchia canariensis Type 2 16S ribosomal RNA gene | | | |
| **EU831094** | Wolbachia endosymbiont of Folsomia candida 16S ribosomal RNA gene | | | |
| **AJ422184** | Wolbachia endosymbiont of Mesaphorura macrochaeta partial 16S rRNA gene | | | |
| **COI** | | | | |
| **DQ345082** | Lucilia bazini cytochrome oxidase subunit I (COI) gene | | | |
| **DQ345084** | Lucilia hainanensis cytochrome oxidase subunit I (COI) gene | | | |
| **DQ453493** | Hemipyrellia ligurriens cytochrome oxidase subunit I (COI) gene | | | |
| **DQ295312** | Calliphora sp. cytochrome c oxidase subunit I (COI) gene | | | |
| **DQ345083** | Hypopygiopsis infumata cytochrome oxidase subunit I (COI) gene | | | |
| **EF531211** | Ornithomya avicularia cytochrome oxidase subunit I (COI) gene | | | |
| **EF531213** | Ornithomya chloropus cytochrome oxidase subunit I (COI) gene | | | |
| **EF531227** | Ornithomya anchineura cytochrome oxidase subunit I (COI) gene | | | |
| **EF531214** | Ornithomya fringillina cytochrome oxidase subunit I (COI) gene | | | |
| **EF531215** | Stenepteryx hirundinis cytochrome oxidase subunit I (COI) gene | | | |
| **EF531212** | Ornithomya biloba cytochrome oxidase subunit I (COI) gene | | | |
| **EF531196** | Crataerina pallida cytochrome oxidase subunit I (COI) gene | | | |
| **KF453419** | Crataerina melbae cytochrome oxidase subunit I gene | | | |
| **KF453424** | Ornithomya fringillina cytochrome oxidase subunit I gene | | | |
| **EF531209** | Melophagus ovinus cytochrome oxidase subunit I (COI) gene | | | |
| **KF453415** | Lipoptena capreoli cytochrome oxidase subunit I gene | | | |
| **EF531204** | Lipoptena cervi cytochrome oxidase subunit I (COI) gene | | | |
| **KF453417** | Lipoptena fortisetosa cytochrome oxidase subunit I gene | | | |
| **KF453418** | Lipoptena sp. OD2013 cytochrome oxidase subunit I gene | | | |
| **EF531207** | Hippobosca rufipes cytochrome oxidase subunit I (COI) gene | | | |
| **EF531208** | Hippobosca equina cytochrome oxidase subunit I (COI) gene | | | |
| **KF453414.** | Hippobosca longipennis cytochrome oxidase subunit I gene | | | |
| **EF531205** | Lipoptena mazamae cytochrome oxidase subunit I (COI) gene | | | |
| **EF531206** | Lipoptena depressa cytochrome oxidase subunit I (COI) gene | | | |
| **JF439541** | Glossina morsitans voucher WVU20110181 cytochrome oxidase subunit I (COI) gene | | | |
| **KF453437** | Glossina fuscipes fuscipes cytochrome oxidase subunit I gene | | | |
| **JQ246707** | Ornithoctona erythrocephala isolate C56 cytochrome c oxidase subunit I (COI) gene | | | |
| **EF531220** | Pseudolynchia sp. P27 cytochrome oxidase subunit I (COI) gene | | | |
| **EF531221** | Ortholfersia minuta cytochrome oxidase subunit I (COI) gene | | | |
| **DQ888274** | Icosta americana isolate 773 cytochrome oxidase subunit I (COI) gene | | | |
| **EF202002** | Icosta nigra isolate NOWD Inigra Isa cytochrome c oxidase subunit I gene | | | |
| **DQ217765** | Ornithomya fringillina isolate Fr2 cytochrome oxidase subunit I (COI) gene | | | |
| **KJ174684** | Ornithophila gestroi voucher MNCN ADN65233 cytochrome oxidase subunit 1 (COI) gene | | | |
| **EU591836** | Glossina morsitans morsitans isolate Gmm LSTM cytochrome oxidase subunit I (COI) gene | | | |
| **EU591835** | Glossina morsitans centralis isolate Gmc IAEA cytochrome oxidase subunit I (COI) gene | | | |
| **GQ255905** | Glossina morsitans submorsitans cytochrome oxidase subunit I (COI) gene | | | |
| **EU591845** | Glossina pallidipes isolate Gpallid 3 cytochrome oxidase subunit I (COI) gene | | | |
| **EU591870** | Glossina fuscipes quanzensis isolate Gfq 6 cytochrome oxidase subunit I (COI) gene | | | |
| **EU591872** | Glossina fuscipes fuscipes isolate Gff IAEA cytochrome oxidase subunit I (COI) gene | | | |
| **HQ387063** | Glossina fuscipes martinii isolate Gfm Kig 10 cytochrome oxidase subunit I (COI) gene | | | |
| **EU591865** | Glossina palpalis palpalis isolate Gpp 20 cytochrome oxidase subunit I (COI) gene | | | |
| **EU591855** | Glossina palpalis gambiensis isolate Gpg 14 cytochrome oxidase subunit I (COI) gene | | | |
| **EU591864** | Glossina pallicera pallicera isolate Gpallic 6 cytochrome oxidase subunit I (COI) gene | | | |
| **EU591844** | Glossina tachinoides isolate Gtac 3 cytochrome oxidase subunit I (COI) gene | | | |
| **EU591834** | Glossina austeni isolate Gaus col RSA cytochrome oxidase subunit I (COI) gene | | | |
| **EU591868** | Glossina medicorum isolate Gmed 3 cytochrome oxidase subunit I (COI) gene | | | |
| **EU591869** | Glossina brevipalpis isolate Gbrev col RSA cytochrome oxidase subunit I (COI) gene | | | |
| **EF531224** | Dipseliopoda setosa cytochrome oxidase subunit I (COI) gene | | | |
| **EF531225** | Basilia boardmani cytochrome oxidase subunit I (COI) gene | | | |
| **KF021535** | Penicillidia oceanica isolate N17 cytochrome c oxidase subunit 1 (COI) gene | | | |
| **KF021534** | Penicillidia leptothrinax isolate J67 cytochrome c oxidase subunit 1 (COI) gene | | | |
| **KF021520** | Penicillidia fulvida isolate N20 cytochrome c oxidase subunit 1 (COI) gene | | | |
| **KF021517** | Nycteribia stylidiopsis isolate B cytochrome c oxidase subunit 1 (COI) gene | | | |
| **KF021492** | Eucampsipoda africana isolate N6 cytochrome c oxidase subunit 1 (COI) gene | | | |
| **KF021504** | Nycteribia schmidlii isolate N3 cytochrome c oxidase subunit 1 (COI) gene | | | |
| **KF021501** | Nycteribia parvula isolate N16 cytochrome c oxidase subunit 1 (COI) gene | | | |
| **KF021500** | Eucampsipoda theodori isolate 8B cytochrome c oxidase subunit 1 (COI) gene | | | |
| **KF021495** | Eucampsipoda madagascarensis isolate J48 cytochrome c oxidase subunit 1 (COI) gene | | | |
| **KF021493** | Eucampsipoda inermis isolate N8 cytochrome c oxidase subunit 1 (COI) gene | | | |
| **KF273783** | Eucampsipoda sundaica isolate KB5 cytochrome oxidase subunit I (COI) gene | | | |
| **KF273782** | Cyclopodia horsfieldi isolate RC24 cytochrome oxidase subunit I (COI) gene | | | |
| **DQ888272** | Paratrichobius longicrus isolate 732 cytochrome oxidase subunit I (COI) gene | | | |
| **DQ888271** | Neotrichobius n. sp. GMB-2006 isolate 72 cytochrome oxidase subunit I (COI) gene | | | |
| **DQ888267** | Megistopoda aranea isolate 736 cytochrome oxidase subunit I (COI) gene | | | |
| **DQ888266** | Neotrichobius delicatus isolate 747 cytochrome oxidase subunit I (COI) gene | | | |
| **DQ888270** | Aspidoptera phyllostomatis isolate 727 cytochrome oxidase subunit I (COI) gene | | | |
| **DQ888269** | Trichobius dugesioides isolate 751 cytochrome oxidase subunit I (COI) gene | | | |
| **DQ888268** | Trichobius lonchophyllae isolate 744 cytochrome oxidase subunit I (COI) gene | | | |
| **DQ888258** | Trichobius joblingi isolate 726 cytochrome oxidase subunit I (COI) gene | | | |
| **DQ888273** | Trichobius major isolate 777 cytochrome oxidase subunit I (COI) gene | | | |
| **DQ888263** | Speiseria ambigua isolate 752 cytochrome oxidase subunit I (COI) gene | | | |
| **KF453436** | Brachytarsina alluaudi cytochrome oxidase subunit I gene, partial cds; mitochondrial | | | |
| **AB632571** | Brachytarsina kanoi mitochondrial gene for cytochrome oxidase subunit I | | | |
| **KF453433** | Penicillidia conspicua cytochrome oxidase subunit I gene | | | |
| **KF453431** | Nycteribia kolenatii cytochrome oxidase subunit I gene | | | |
| **KF453429** | Nycteribia schmidlii scotti cytochrome oxidase subunit I gene | | | |
| **KF453428** | Nycteribia schmidlii schmidlii cytochrome oxidase subunit I gene | | | |
| **AB632558** | Nycteribia pleuralis mitochondrial gene for cytochrome oxidase subunit I, isolate: NyPl6 | | | |
| **AB632552** | Nycteribia pygmaea mitochondrial gene for cytochrome oxidase subunit I, isolate: NyPy5 | | | |
| **AB632547** | Nycteribia allotopa mitochondrial gene for cytochrome oxidase subunit I, isolate: NyAl11 | | | |
| **AB632538** | Basilia rybini mitochondrial gene for cytochrome oxidase subunit I, isolate: BaRy1 | | | |
| **AB632537** | Basilia truncata mitochondrial gene for cytochrome oxidase subunit I, isolate: BaTr8 | | | |
| **AB632570** | Phthiridium hindlei mitochondrial gene for cytochrome oxidase subunit I, isolate: PhHi3 | | | |
| **AB632565** | Penicillidia monoceros mitochondrial gene for cytochrome oxidase subunit I, isolate: PeMo2 | | | |
| **AB632563** | Penicillidia jenynsii mitochondrial gene for cytochrome oxidase subunit I, isolate: PeJe7 | | | |
| **KF725678** | Olfersia sp. TR HMB1 cytochrome oxidase subunit 1 gene | | | |
| **KC700583** | Olfersia aenescens isolate HIPP167 cytochrome oxidase subunit 1 gene | | | |
| **KC700578** | Olfersia spinifera isolate HIPP72 cytochrome oxidase subunit 1 gene | | | |
| **EF531223** | Ornithoctona sp. P-20 cytochrome oxidase subunit I (COI) gene | | | |
| **EF531198** | Glossina austeni cytochrome oxidase subunit I (COI) gene | | | |
| **EF531202** | Glossina palpalis cytochrome oxidase subunit I (COI) gene | | | |
| **EF531199** | Glossina brevipalpis cytochrome oxidase subunit I (COI) gene | | | |
| **EF531226** | Glossina fuscipes cytochrome oxidase subunit I (COI) gene | | | |
| **EF531203** | Glossina swynnertoni cytochrome oxidase subunit I (COI) gene | | | |
| **KC840682** | Lucilia sericata isolate sgly6LW2 cytochrome oxidase subunit I (COI) gene | | | |
| **JQ807056** | Pollenia rudis voucher AZ63 cytochrome oxidase subunit I (COI) gene | | | |
| **KF919047** | Protophormia terraenovae voucher NICC0358 11615010 cytochrome oxidase subunit 1 (COI) gene | | | |
| **KM571920** | Musca domestica voucher 08BBDIP-0873 cytochrome oxidase subunit 1 (COI) gene | | | |
| **KJ167551** | Sarcophaga crassipalpis voucher BIOUG03032-B12 cytochrome oxidase subunit 1 (COI) gene | | | |
| **16S** | | | | |
| **EF531121** | Stenepteryx hirundinis 16S ribosomal RNA gene | | | |
| **EF531119** | Ornithomya biloba 16S ribosomal RNA gene | | | |
| **EF531107** | Crataerina pallida 16S ribosomal RNA gene | | | |
| **EF531118** | Ornithomya avicularia 16S ribosomal RNA gene | | | |
| **EF531120** | Ornithomya chloropus 16S ribosomal RNA gene | | | |
| **EF531103** | Pseudolynchia sp. P-27 16S ribosomal RNA gene | | | |
| **AB632589** | Lipoptena fortisetosa mitochondrial genes for 16S ribosomal RNA | | | |
| **AF322437** | Lipoptena cervi 16S ribosomal RNA gene | | | |
| **EF531104** | Melophagus ovinus 16S ribosomal RNA gene | | | |
| **EF531123** | Ortholfersia minuta 16S ribosomal RNA gene | | | |
| **EF531116** | Hippobosca equina 16S ribosomal RNA gene | | | |
| **EF531115** | Hippobosca rufipes 16S ribosomal RNA gene | | | |
| **EF531112** | Glossina palpalis 16S ribosomal RNA gene | | | |
| **EF531126** | Glossina fuscipes 16S ribosomal RNA gene | | | |
| **EF531110** | Glossina morsitans 16S ribosomal RNA gene | | | |
| **EF531113** | Glossina swynnertoni 16S ribosomal RNA gene | | | |
| **EF531111** | Glossina pallidipes 16S ribosomal RNA gene | | | |
| **EF531108** | Glossina austeni 16S ribosomal RNA gene | | | |
| **AB632588** | Brachytarsina kanoi mitochondrial genes for 16S ribosomal RNA | | | |
| **DQ133049** | Megastrebla nigriceps '1' Di138 16S ribosomal RNA gene | | | |
| **DQ133024** | Megastrebla parvior parvior 16S ribosomal RNA gene | | | |
| **EF531109** | Glossina brevipalpis 16S ribosomal RNA gene | | | |
| **AB632587** | Phthiridium hindlei mitochondrial genes for 16S ribosomal RNA | | | |
| **AB632581** | Nycteribia pleuralis mitochondrial genes for 16S ribosomal RNA | | | |
| **AB632579** | Nycteribia pygmaea mitochondrial genes for 16S ribosomal RNA | | | |
| **AB632577** | Nycteribia allotopa mitochondrial genes for 16S ribosomal RNA | | | |
| **DQ133026** | Phtiridium fraterna 16S ribosomal RNA gene | | | |
| **AB632575** | Basilia rybini mitochondrial genes for 16S ribosomal RNA | | | |
| **DQ133037** | Basilia coronata indivisa 16S ribosomal RNA gene | | | |
| **AB632574** | Basilia truncata mitochondrial genes for 16S ribosomal RNA | | | |
| **KJ131412** | Nycteribia schmidlii haplotype 32 16S ribosomal RNA gene | | | |
| **AB632585** | Penicillidia monoceros mitochondrial genes for 16S ribosomal RNA | | | |
| **AB632583** | Penicillidia jenynsii mitochondrial genes for 16S ribosomal RNA | | | |
| **AF322435** | Penicillidia sp. VH-2001 16S ribosomal RNA gene | | | |
| **EF531125** | Dipseliopoda setosa 16S ribosomal RNA gene | | | |
| **EF531124** | Penicillidia fulvida 16S ribosomal RNA gene | | | |
| **DQ133030** | Basilia forcipata 16S ribosomal RNA gene | | | |
| **DQ133025** | Basilia corynorhini 16S ribosomal RNA gene | | | |
| **DQ133047** | Strebla mirabilis 16S ribosomal RNA gene | | | |
| **DQ133052** | Trichobius longipes 16S ribosomal RNA gene | | | |
| **DQ133023** | Trichobius hirsutulus 16S ribosomal RNA gene | | | |
| **DQ133046** | Trichobius major '2' Di149 16S ribosomal RNA gene | | | |
| **DQ133044** | Trichobius corynorhini 16S ribosomal RNA gene | | | |
| **DQ133050** | Trichobius yunkeri '2' Di152 16S ribosomal RNA gene | | | |
| **DQ133029** | Trichobius caecus 16S ribosomal RNA gene | | | |
| **DQ133035** | Trichobius dugesii 16S ribosomal RNA gene | | | |
| **DQ133036** | Trichobius intermedius 16S ribosomal RNA gene | | | |
| **DQ133051** | Trichobius parasiticus 16S ribosomal RNA gene | | | |
| **DQ133028** | Trichobius diaemi 16S ribosomal RNA gene | | | |
| **DQ133038** | Dipseliopoda biannulata 16S ribosomal RNA gene | | | |
| **DQ133041** | Eucampsipoda inermis 16S ribosomal RNA gene | | | |
| **DQ133034** | Eucampsipoda penthetoris 16S ribosomal RNA gene | | | |
| **DQ133048** | Ascodipteron sp. Di137 16S ribosomal RNA gene | | | |
| **DQ133042** | Ascodipteron phyllorhinae 16S ribosomal RNA gene | | | |
| [**KC347601**](http://www.ncbi.nlm.nih.gov/nucleotide/451899300?report=genbank&log$=nucltop&blast_rank=1&RID=3HXDP9WK01R) | [Musca domestica 16S ribosomal RNA gene](http://blast.ncbi.nlm.nih.gov/Blast.cgi#alnHdr_451899300) | | | |
| [**JN226657**](http://www.ncbi.nlm.nih.gov/nucleotide/343170809?report=genbank&log$=nucltop&blast_rank=1&RID=3HXY7PHR015) | [Stomoxys indicus 16S ribosomal RNA gene](http://blast.ncbi.nlm.nih.gov/Blast.cgi#alnHdr_343170809) | | | |
| [**JN226656**](http://www.ncbi.nlm.nih.gov/nucleotide/343170808?report=genbank&log$=nucltop&blast_rank=1&RID=3HY0EKBR014) | [Graphomya tienmushanensis 16S ribosomal RNA gene](http://blast.ncbi.nlm.nih.gov/Blast.cgi#alnHdr_343170808) | | | |
| [**JN226653**](http://www.ncbi.nlm.nih.gov/nucleotide/343170805?report=genbank&log$=nucltop&blast_rank=1&RID=3HY3UFHW014) | [Lispe quaerens 16S ribosomal RNA gene](http://blast.ncbi.nlm.nih.gov/Blast.cgi#alnHdr_343170802) | | | |
| [**GQ409146**](http://www.ncbi.nlm.nih.gov/nucleotide/258537775?report=genbank&log$=nucltop&blast_rank=1&RID=3HYPR4J7014) | Pollenia rudis voucher RMBR:103623 16S ribosomal RNA gene | | | |
| [**JQ307754**](http://www.ncbi.nlm.nih.gov/nucleotide/409154283?report=genbank&log$=nucltop&blast_rank=1&RID=3HYTFK5F014) | [Protophormia terraenovae 16S ribosomal RNA gene](http://blast.ncbi.nlm.nih.gov/Blast.cgi#alnHdr_409154283) | | | |
| [**GU145217**](http://www.ncbi.nlm.nih.gov/nucleotide/270741285?report=genbank&log$=nucltop&blast_rank=1&RID=3HYVJDRZ014) | Lucilia sericata isolate CSU09100410A1 16S ribosomal RNA gene | | | |
| [**AF468210**](http://www.ncbi.nlm.nih.gov/nucleotide/18698660?report=genbank&log$=nucltop&blast_rank=1&RID=3HZBNN5V01R) | Sarcophaga crassipalpis 16S ribosomal RNA gene | | | |
| **EF** | | | | |
| [**AY123346**](http://www.ncbi.nlm.nih.gov/nucleotide/21629704?report=genbank&log$=nucltop&blast_rank=1&RID=3HYKG65N014) | Musca domestica 16S ribosomal RNA gene | | | |
| **JF439518** | Glossina morsitans voucher WVU2011-018-1 elongation factor 1-alpha (EF1alpha) gene | | | |
| **AFB71000** | elongation factor 1-alpha, partial [Glossina morsitans] | | | |
| **JF439523** | Sarcophaga crassipalpis voucher WVU2011-018-7 elongation factor 1-alpha (EF1alpha)**gene** | | | |
| **JF439528** | Lucilia sericata voucher WVU2009-017-20 elongation factor 1-alpha EF1alpha) **gene** | | | |
| **JF439529** | Hypopygiopsis infumata voucher WVU2011-018-13 elongation factor 1-alpha (EF1alpha)**gene** | | | |
| **JF439530** | Phormia regina voucher WVU2009-017-18 elongation factor 1-alpha (EF1alpha) **gene** | | | |
| **FR719265** | Pollenia rudis**partial**EF-1a**gene**for elongation factor 1 alpha protein | | | |
| [**DQ657113**](http://www.ncbi.nlm.nih.gov/nucleotide/109894483?report=genbank&log$=nucltop&blast_rank=1&RID=3HYEGE5U014) | [Musca domestica elongation factor-1 alpha (Ef1a) gene](http://blast.ncbi.nlm.nih.gov/Blast.cgi#alnHdr_109894483) | | | |
| **Mitochondrial genomes** | | | | |
| **JQ862475** | Liriomyza sativae mitochondrion, complete genome | | | |
| **JX913762** | Rutilia goerlingiana mitochondrion, complete genome | | | |
| **KM200723** | Musca domestica mitochondrion, complete genome | | | |
| **KM200724** | Scathophaga stercoraria mitochondrion, complete genome | | | |
| **KP091687** | Sarcophaga melanura mitochondrion, complete genome | | | |
| **KP901269** | Euryomma sp. SMD-2015 mitochondrion, partial genome | | | |
| **NC006378** | Dermatobia hominis mitochondrion, complete genome | | | |
| **NC019639** | Calliphora vicina mitochondrion, complete genome | | | |
| **NC028226** | Delia antiqua mitochondrion, complete genome | | | |
| **NC028518** | Drosophila formosana mitochondrion, complete genome | | | |
| **ftp://ftp.ncbi.nih.gov/genomes/genbank/invertebrate/Glossina_morsitans/latest_assembly_versions/GCA_001014515.1_ASM101451v1** | | | Glossina morsitans, whole genome shotgun sequence | |

Baldo L., Dunning Hotopp JC., Jolley KA., Bordenstein SR., Biber SA., Choudhury RR., Hayashi C., Maiden MCJ., Tettelin H., Werren JH. 2006. Multilocus sequence typing system for the endosymbiont Wolbachia pipientis. *Applied and environmental microbiology* 72:7098–110. DOI: 10.1128/AEM.00731-06.

Brown J., Pellmyr O., Thompson J., Harrison R. 1994. Phylogeny of Greya (Lepidoptera: Prodoxidae), based on nucleotide sequence variation in mitochondrial cytochrome oxidase I and II: congruence with morphological data. *Mol. Biol. Evol.* 11:128–141.

Hypša V., K\vr’ižek J. 2007. Molecular Evidence for Polyphyletic Origin of the Primary Symbionts of Sucking Lice (Phthiraptera, Anoplura). *Microbial Ecology* 54:242–251. DOI: 10.1007/s00248-006-9194-x.

Moran NA., Kaplan ME., Gelsey MJ., Murphy TG., Scholes EA. 1999. Phylogenetics and evolution of the aphid genus Uroleucon based on mitochondrial and nuclear DNA sequences. *Systematic Entomology* 24:85–93. DOI: 10.1046/j.1365-3113.1999.00076.x.

O’Neill SL., Giordano R., Colbert AM., Karr TL., Robertson HM. 1992. 16S rRNA phylogenetic analysis of the bacterial endosymbionts associated with cytoplasmic incompatibility in insects. *Proceedings of the National Academy of Sciences* 89:2699–2702. DOI: 10.1073/pnas.89.7.2699.

Petersen FT., Meier R., Kutty SN., Wiegmann BM. 2007. The phylogeny and evolution of host choice in the Hippoboscoidea (Diptera) as reconstructed using four molecular markers. *Molecular phylogenetics and evolution* 45:111–22. DOI: 10.1016/j.ympev.2007.04.023.
