# Supplementary figures and images for: Arsenophonus and Sodalis replacements shape evolution of symbiosis in louse flies
Source: PeerJ. 2017 Dec 11;5:e4099. doi: 10.7717/peerj.4099 (PMC5729840; doi:10.7717/peerj.4099)

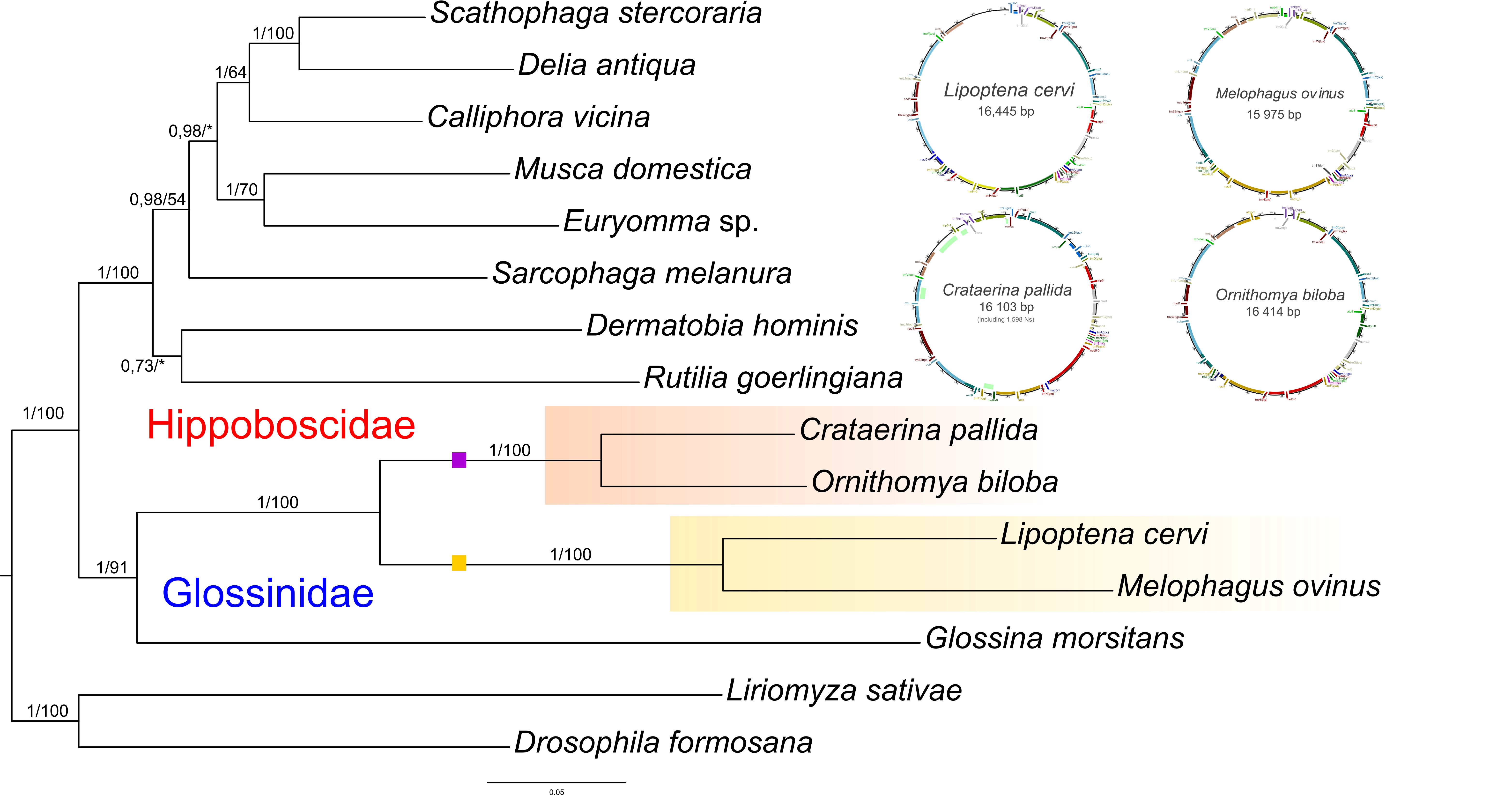

Supplement: Figure S1 — Phylogeny of Hippoboscoidea based on 15 mitochondrial genes. It includes figures of four mitochondrial genomes assembled and annotated in this study which were also used for phylogeny reconstruction. [file peerj-05-4099-s004.png]
